# Azaphilones from the Marine Sponge-Derived Fungus *Penicillium sclerotiorum* OUCMDZ-3839

**DOI:** 10.3390/md17050260

**Published:** 2019-04-30

**Authors:** Qian Jia, Yuqi Du, Chen Wang, Yi Wang, Tonghan Zhu, Weiming Zhu

**Affiliations:** 1 Key Laboratory of Marine Drugs, Ministry of Education of China, School of Medicine and Pharmacy, Ocean University of China, Qingdao 266003, China; jiaqianxiaoyugan@126.com (Q.J.); mystagedu@foxmail.com (Y.D.); wangchen133wc@163.com (C.W.); wangyi0213@ouc.edu.cn (Y.W.); sdueduzth@126.com (T.Z.); 2 Open Studio for Druggability Research of Marine Natural Products, Laboratory for Marine Drugs and Bioproducts, Pilot National Laboratory for Marine Science and Technology (Qingdao), Qingdao 266003, China; 3 College of Computer Science and Engineering, Shandong University of Science and Technology, Qingdao 266590, China

**Keywords:** azaphilones, anti-virus, anti-α-glycosidase, *Paratetilla* sp., *Penicillium sclerotiorum*

## Abstract

Four new azaphilones, sclerotiorins A–D (**1**–**4**), as well as the dimeric sclerotiorin E (**5**) of which we first determined its absolute configuration, and 12 known analogues (**5**–**16**) were isolated from the fermentation broth of *Penicillium sclerotiorum* OUCMDZ-3839 associated with a marine sponge *Paratetilla* sp.. The new structures, including absolute configurations, were elucidated by spectroscopic analyses, optical rotation, ECD spectra, X-ray single-crystal diffraction, and chemical transformations. Compounds **11** and **14** displayed significant inhibitory activity against α-glycosidase, with IC_50_ values of 17.3 and 166.1 μM, respectively. In addition, compounds **5**, **7**, **10**, **12**–**14,** and **16** showed moderate bioactivity against H1N1 virus.

## 1. Introduction

Marine fungi are important sources of bioactive natural products (NPs) [1]. Accordingly, more than 3000 NPs were discovered from marine fungi in the past decades, accounting for 27% of all the marine-derived NPs [2,3,4,5,6,7]. Azaphilones are a class of biologically active metabolites of fungi and have been reported to display antimicrobial, antiviral, antioxidant, cytotoxic, nematocidal, and anti-inflammatory bioactivities [8,9]. So far, over 430 azaphilones derived from both marine and terrestrial fungi have been reported, such as the cytotoxic chaetomugilins A–O [10,11,12,13] and isochromophilones A–F [14], anti-bacterial penicilones A–H [15,16] and pleosporalones B–C [17], anti-inflammatory monapilol A–D [18], and others.

**Figure 1:**
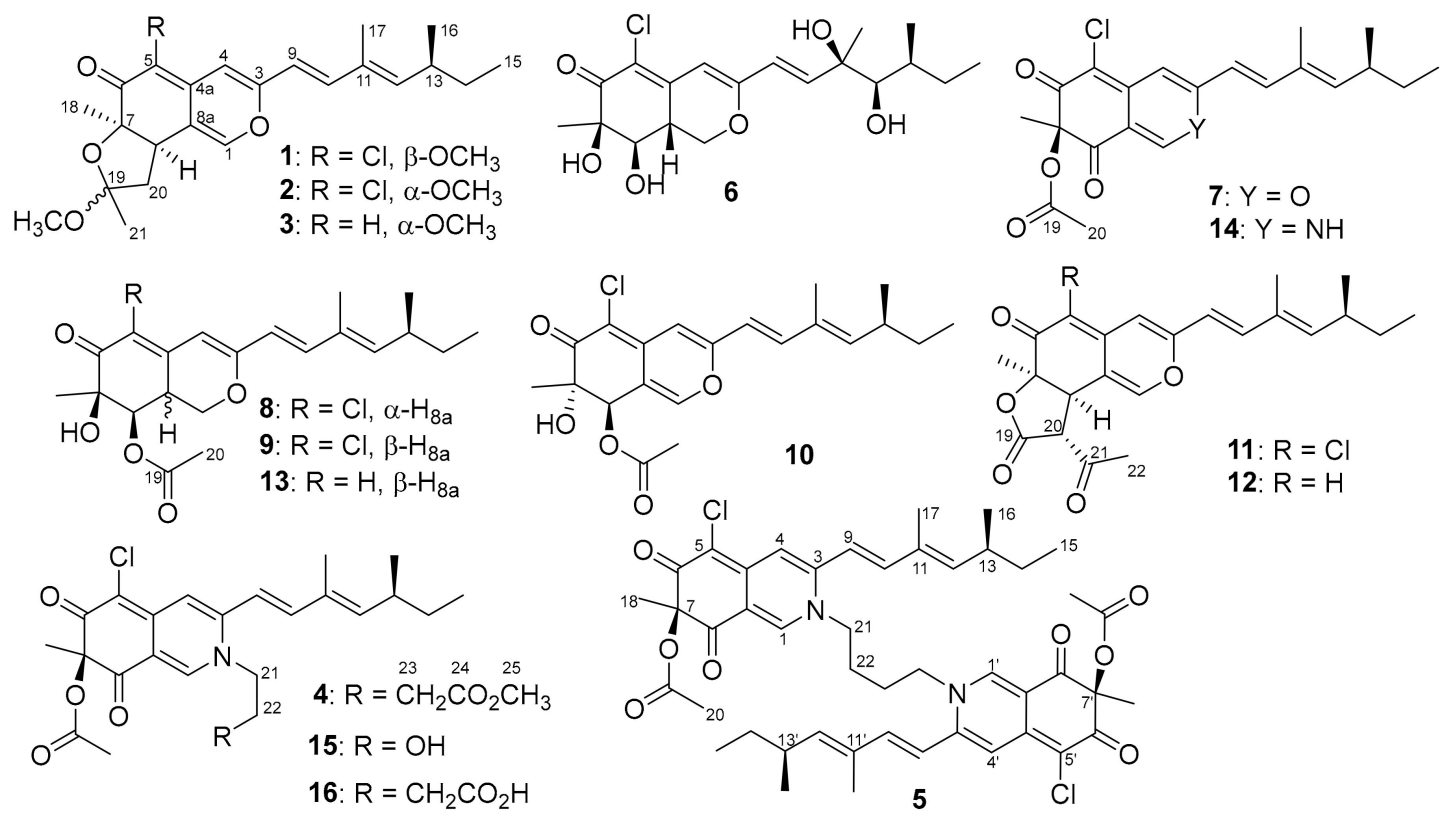
Structures of compounds **1**–**16**.

The chemical investigation of a *Paratetilla* sp. sponge-derived fungus, *Penicillium sclerotiorum* OUCMDZ-3839, led to the identification of four new azaphilones, called sclerotiorins A–D (**1**–**4**), as well as 12 known analogues (Figure 1), sclerotiorin E (**5**) [19], geumsanol G (**6**) [20,21], (+)sclerotiorin (**7**) [22,23,24,25], isochromophilone I (**11**) [26], IV (**9**) [27], VI (**15**) [27,28], VIII (**8**) [29], and IX (**16**) [28], TL-1-monoactate (**10**) [30], ochrephilone (**12**) [24,31], 8-acetyldechloroisochromophilone III (**13**) [32], and scleratioramine (**14**) [24,33]. Although the composition of sclerotiorin E (**5**) was previously reported, its absolute configuration is determined in the current work for the first time. Compounds **5**, **7**, **10**, **12**–**14,** and **16** showed stronger antiviral activity against H1N1 in the MDCK cell line than the positive control ribavirin. Furthermore, compounds **11** and **14** displayed significant inhibitory activity against *α*-glycosidase, with IC_50_ values of 17.3 and 166.1 μM, respectively.

## 2. Results and Discussion

**Figure 2:**
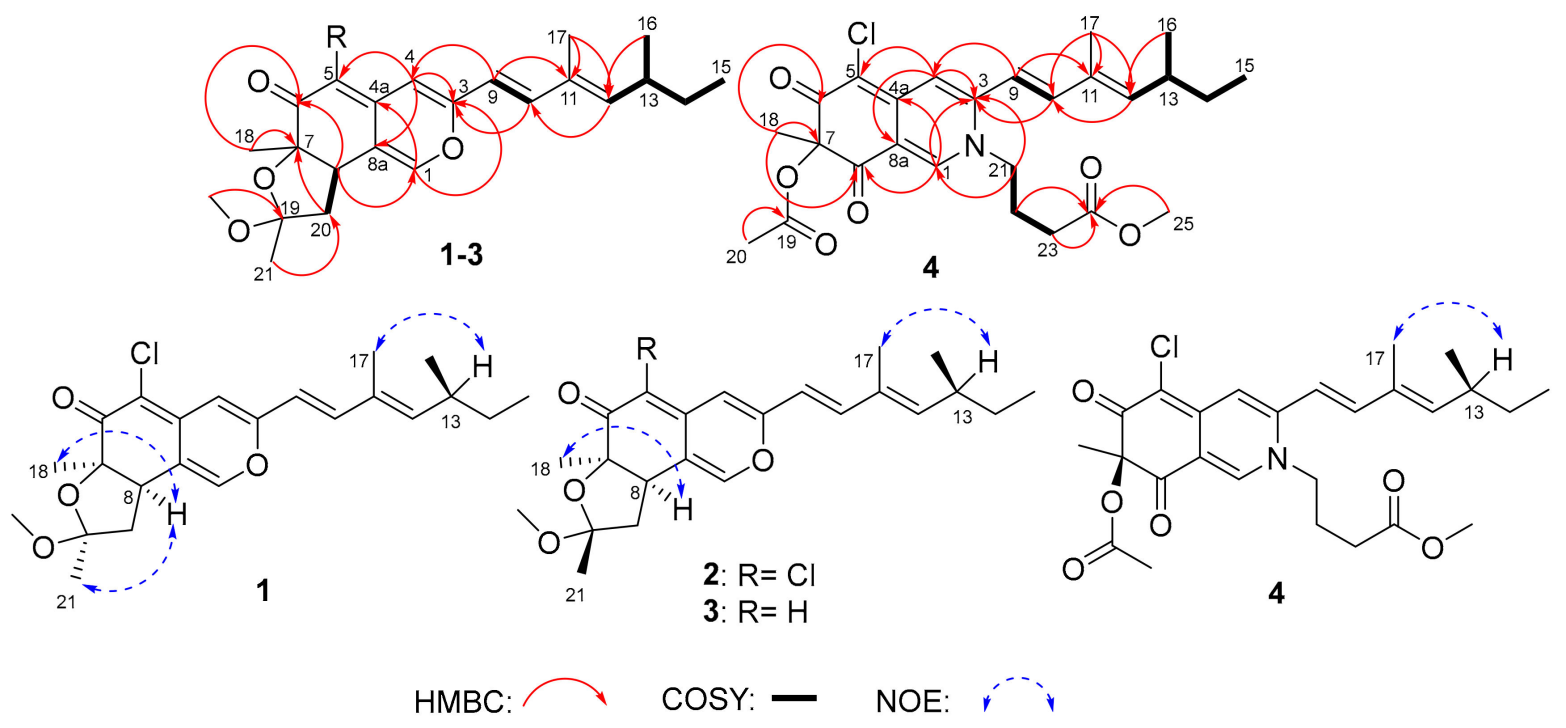
Key COSY, HMBC, and Nuclear Overhauser Effect (NOE) correlations of compounds **1**–**4**.

Sclerotiorin A (**1**) was obtained as a yellow amorphous powder. The molecular formula of **1** was determined to be C_23_H_29_O_4_Cl by the HRESIMS (high resolution electrospray ionization mass spectroscopy) peak at *m*/*z* 405.1834 [M + H]^+^ (Figure S1), with the 1:3 chlorine isotope peaks. ^1^H (Table 1, Figure S2), ^13^C (Table 2, Figure S3) combined with DEPT (distortionless enhancement by polarization transfer, Figure S4) and HSQC (heteronuclear single-quantum correlation, Figure S5) NMR data of **1** revealed the presence of 5 singlet methyls, 1 methoxy, two methylenes, 2 sp^3^ methines, 2 heteroatom-bonded sp^3^ non-protonated carbons, 10 olefinic/aromatic carbons, and 1 carbonyl. The HMBC (heteronuclear multiple bond correlation, Figure 2 and Figure S7) from H-1 to C-3 and C-4a, H-4 to C-3, C-5, and C-8a, H-8 to C-1, C-4a, C-6, and C-7 established the core skeleton of azaphilones.^8^ Moreover, the COSY (correlation spectroscopy) cross peaks (Figure 2 and Figure S6) from H-9 to H-10 and from H-13 to H-12, H-14, H-16, then from H-14 to H-15, along with the HMBC correlations from H-9 to C-11, H-10 to C-17 and C-12, H-12 to C-10 and C-17 demonstrated the presence of the common side chain of azaphilones [8]. The linkage of the unsaturated side chain to C-3 was demonstrated by the HMBC correlations from H-9 to C-3 and C-4 along with that from H-10 to C-3. Additionally, the other HMBC correlations from H-20a to C-7, H-20b to C-19, C-8a ,and C-8, H-*β*-OCH_3_ to C-19, and H-21 to C-20 revealed a furan nucleus. The COSY correlations from H-8 to H-20, as well as the HMBC correlations from H-20 to C-7 and C-8, confirmed the connection mode of the furan nucleus to the fused pyrone–quinone core skeleton. Thus, the constitution of sclerotiorin A (**1**) was determined.

Sclerotiorin B (**2**) was obtained as a yellow amorphous powder. HRESIMS gave the peak of *m*/*z* 405.1836 [M + H]^+^ (Figure S10) and the chlorine isotope peaks; consequently, the molecular formula was determined to be C_23_H_29_O_4_Cl, the same as that of **1**. The NMR data of **2** ( Table 1; Table 2, Figures S11–S14) were similar to those of **1**, except that C-8, C-19, and C-20 were shielded and shifted from *δ*_C_ 43.4, 106.0, and 45.8 of **1** to *δ*_C_ 42.7, 105.4, and 44.2 of **2**, respectively. The chemical shift changes may be triggered by the difference of the stereochemistry on C-19. Accordingly, compound **2** was identified as a 19-epimer of compound **1**, which was further confirmed by the 2D NMR data (Figure 2, Figure S15 and Figure S16).

Sclerotiorin C (**3**) was also obtained as a yellow powder. The molecular formula was deduced to be C_23_H_30_O_4_ by the *m*/*z* 371.2218 [M + H]^+^ of the HRESIMS (Figure S18), without the chlorine isotope peaks. The NMR data (Table 1 and Table 2, Figures S19–S23) and optical rotation of **3** were similar to those of **2**, except for the expected extra CH signal (*δ*_C/H_ 106.1/5.23) on C-5 indicating that the chlorine atom at C-5 in **3** was substituted by a hydrogen atom. The key HMBC correlations (Figure S24) from H-5 (*δ*_H_ 5.23) to C-7 (*δ*_C_ 83.1), C-8a (*δ*_C_ 116.4), and C-4 (*δ*_C_ 107.7) confirmed the position of the extra H-5. Thus, **3** was identified as the dechlorinated derivative of **2**, named sclerotiorin C.

Sclerotiorin D (**4**) was a red powder after purification. The peak *m*/*z* 490.1993 [M + H]^+^ of HRESIMS (Figure S26) indicated the molecular formula was C_26_H_32_O_6_NCl. When comparing the NMR data (Table 1 and Table 2, Figures S27–S30) of compound **4** to those of **16** [28], the most obvious differences were that compound **4** had one more methyl group (*δ*_C/H-25_ 52.2/3.70), and the carbonyl carbon C-24 (*δ*_C_ 172.6) was deshielded. Consequently, compound **4** was supposed to be the methyl ester derivative of **16**. A further analysis of 1D and 2D NMR confirmed the chemical composition of compound **4** (Figure 2, Figure S31 and Figure S32).

**Figure 3:**
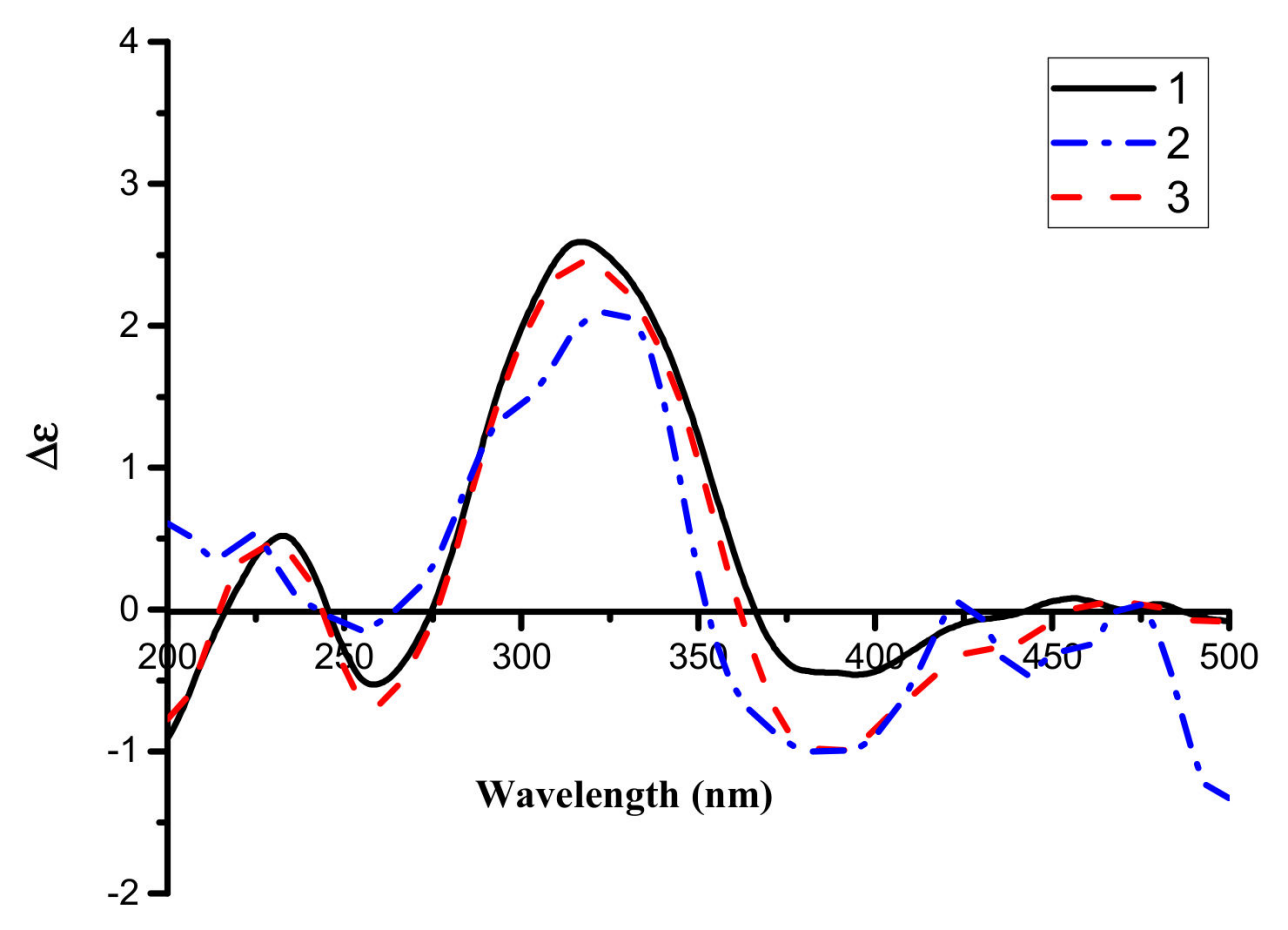
Measured ECD curves of compounds **1**–**3**.

In all new compounds **1**–**4**, the large *J* value between H-9 and H-10 (Table 2) and the NOESY (nuclear Overhauser enhancement spectroscopy) correlations of H-17/H-13 suggested the *E*-type of Δ^9^ and Δ^11^ double bonds (Figure 2, Figures S8, S17, and Figure S25). According to the common Cotton effect of azaphilones established by Steyn and Vleggaar [25,34], the absolute configuration of C-7 in **1**–**3** was suggested to be *R* from the positive Cotton effects at 317 nm (Δ*ε* + 2.59, **1**), 317 nm (Δ*ε* + 2.45, **2**), and 324 nm (Δ*ε* + 2.09, **3**) (Figure 3), which were consistent with those reported for isochromophilones C (314 nm, Δ*ε* + 2.85) and D (314 nm, Δ*ε* + 3.99) [14]. Then, key NOE (Nuclear Overhauser Effect) enhancements of H-18 (*δ*_H_ 1.19) and H-21 (*δ*_H_ 1.35) were observed after irradiation of H-8 (*δ*_H_ 3.23) in **1** (Figure 2 and Figure S9). However, only the H-8/H-18 correlations were observed on the NOESY spectra of **2** and **3**, indicating the opposite configurations of C-19. Therefore, the absolute configurations of sclerotiorin A–C (**1**–**3**) were determined to be 7*R*/8*R*/19*S*, 7*R*/8*R*/19*R,* and 7*R*/8*R*/19*R*, respectively. The absolute configurations of C-13 in **1**–**3** were suggested as *S* by the common biosynthetic pathway of the aliphatic side chain in reported azaphilones [9,14], which were determined by X-ray single-crystal diffraction [35], hydrolysis [36], or ECD (electronic circular dichroism) calculation [14].

**Scheme 1:**
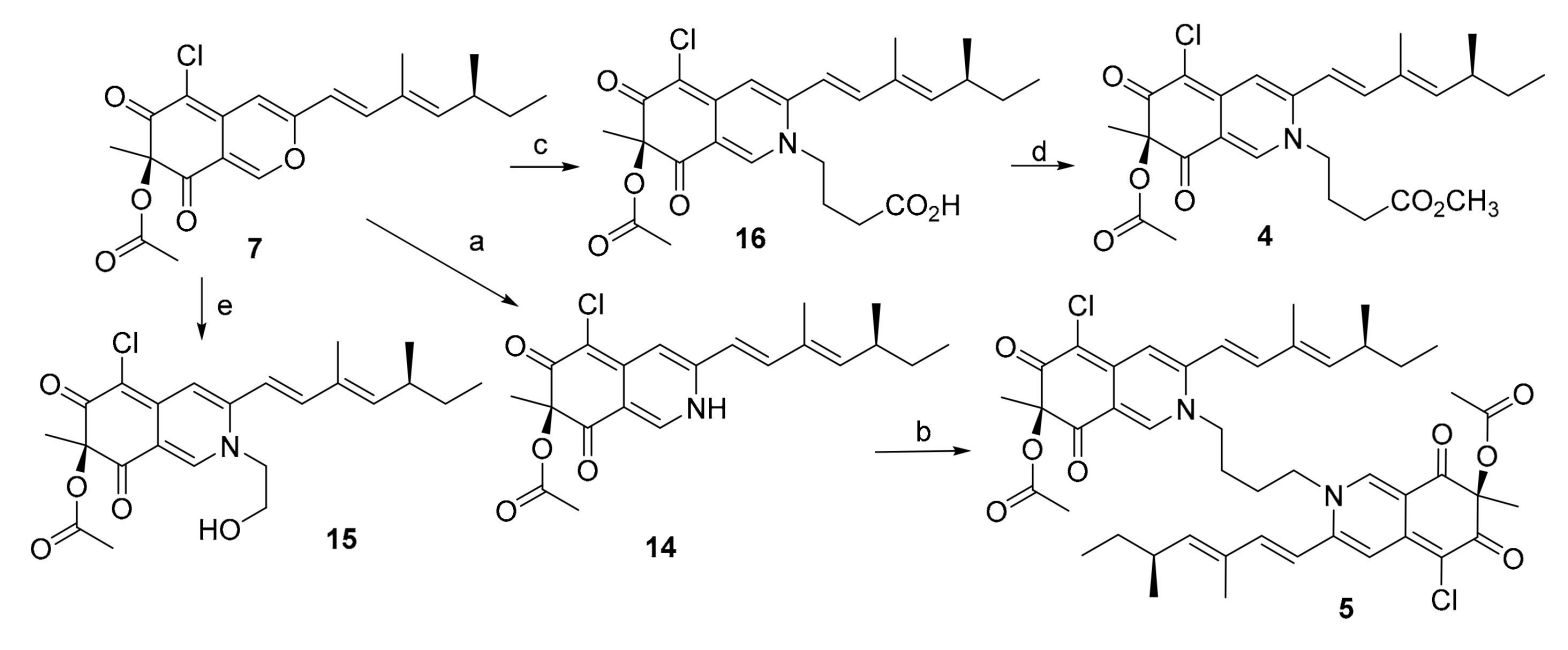
Chemical transformation of **7** to **4**, **5,** and **14**–**16**. Reagents and conditions: a. NH_4_OAc, MeOH, THF (tetrahydrofuran), 17h, r.t (room temperature), 40.1% yield; b. 1,4-diiodobutane, K_2_CO_3_, acetone, 7d, 50 °C, 3.3% yield; c. aminobutyric acid, DMF (*N*,*N*-dimethylformamide), N_2_, 5h, r.t, 92.7% yield; d. CH_3_I, K_2_CO_3_, acetone, N_2_, 24h, r.t, 94.1% yield; e. aminoethanol, CH_2_Cl_2_, N_2_, 24h, r.t, 92.4% yield.

**Figure 4:**
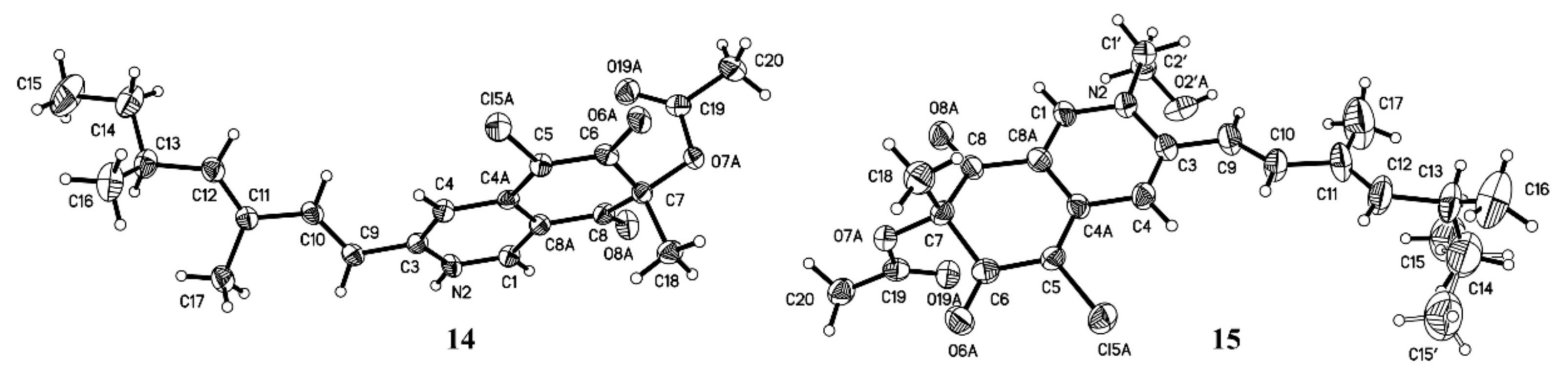
ORTEP (Oak Ridge Thermal-Ellipsoid Plot) drawing of compounds **14** and **15** (Mo Kα).

Taking into account the structural similarity of **14** with **4**, **5**, **7**, **15,** and **16**, the absolute configurations of these compounds could be resolved by chemical correlation if a single crystal of **14** could be obtained. Fortunately, a single crystal of compound **14** was obtained, and the X-ray diffraction (Figure 4) clearly determined the absolute configuration of **14** as 7*R* and 13*S* from the absolute structure parameter of 0.01(2). Thus, a series of reactions were carried out using (+)-sclerotiorin (**7**) as a raw material (Scheme 1) [37]. Compounds **14**–**16** were directly produced after the reaction of **7** with ammonium acetate, aminoethanol, and 3-aminobutyric acid, while compounds **4** and **5** resulted from the reactions of **16** and **14** with iodomethane and 1,4-diiodobutane (Figure S39), respectively. The synthetic compounds **4**, **5,** and **14**–**16** were identified as the natural ones by the same retention times in their co-HPLC profiles, as shown in Figure S37. In addition, compounds **4**, **5,** and **14**–**16** displayed similar ECD (Figure S38) and the same sign of specific rotation. Therefore, compounds **4**, **5,** and **14**–**16** had the same (7*R*,13*S*) configurations.

Sclerotiorin E (**5**) was obtained as a red powder. The HRESIMS peak *m*/*z* 833.3350 [M + H]^+^ indicated the molecular formula was C_46_H_54_O_8_N_2_Cl_2_ (Figure S33). Although the constitution of **5** [19] was identified to be the same as that reported by NMR (Table S1 and S2, Figures S34–S37), the absolute configuration has not been resolved yet. Expectedly, compound **5** was the dimer of **14** linked by a 1,4-butylidene bridge. As shown in Scheme 1, compound **5** could be semi-synthesized from **14**. Thus, the absolute configuration of compound **5** that we named sclerotiorin E was determined to be (7*R*, 7′*R*, 13*S*, 13′*S*) for the first time.

The anti-H1N1-virus activity of compounds **1**–**16** were examined in the MDCK cell line by the CPE (cytopathic effect) + MTT (3-(4,5-dimethyl-2-thiazolyl)-2,5-diphenyl-2-*H*-tetrazolium bromide) method [38,39]. Compounds **5**, **7**, **10**, **12**, **13**, **14,** and **16** showed better inhibitory activity on H1N1 than the positive control (ribavirin), with IC_50_ values ranging from 78.6 to 156.8 μM (Table 3). The results displayed the structure–activity relationships of these azaphilones against the H1N1 virus: the chlorine atom at C-5 was not necessary for the activity, the dimer had a stronger anti-viral activity than the monomer, replacement of the oxygen atom connected to C-1 by a nitrogen atom did not affect the anti-H1N1 activity. Furthermore, the *α*-glycosidase inhibitory activity of compounds **1**–**16** was assayed by the PNPG (*p*-nitrophenyl *β*-D-glucopyranoside) method [40]. Compounds **11** and **14** displayed good activity against *α*-glycosidase, with IC_50_ values of 17.3 and 166.1 μM, respectively (acarbose, 1.1 mM).

## 3. Experimental Section

### 3.1. General Experimental Procedures

All the NMR spectra were recorded on a JEOLJN M-ECP 600 spectrometer (JEOL, Tokyo, Japan) or a Bruker Advance 500 spectrometer (Bruker, Fällanden, Switzerland) used TMS (tetramethylsilane) as internal standard. ^1^H chemical shifts were referenced to the residual CDCl_3_ or DMSO-*d*_6_ signal (*δ*7.26 and 2.50 ppm, respectively). ^13^C chemical shifts were referenced to the CDCl_3_ or DMSO-*d*6 solvent peak (*δ*77.16 or 39.52 ppm, respectively). Optical rotations were measured on a JASCO P-1020 digital polarimeter (JASCO Corporation, Tokyo, Japan). ECD data were acquired on a JASCO J-815 spectropolarimeter (JASCO Corporation, Tokyo, Japan). HRESIMS spectra were collected using the Q-TOF ULTIMA GLOBAL GAA076 LC mass spectrometer (Waters Asia Ltd., Singapore). Semi-preparative HPLC was performed using an ODS (octadecylsilyl) column (YMC-pack ODS-A, 10 × 250 mm, 5 μm, 4 mL/min). TLC was performed on plates precoated with silica gel GF_254_ (10–40 μm, Qingdao Marine Chemical Factory, Qingdao, China). Column chromatography (CC) was carried by silica gel GF_254_ and Sephadex LH-20 (Amersham Biosciences, Uppsala, Sweden). Vacuum–liquid chromatography (VLC) utilized silica gel H (Qingdao Marine Chemical Factory, Qingdao, China).

### 3.2. Fungal Material and Fermentation

The fungus OUCMDZ-3839 was isolated from *Paratetilla* sp. sponges collected from Xisha Island in November 2011. The sponges were firstly clipped, then ground and suspended in sterile distilled water. The suspension was spread on a seawater starch (SWS) agar plate (10 g starch, 1 g protein powder, 20 g agar per liter of sea water). After culturing at 28 °C for several days, the single colony was purified on a potato dextrose agar plate (PDA, 200 g potato, 20 g glucose, 20 g agar per liter of tap water) and maintained at −80 °C. After culture on the PDA plate at 28 °C for 4 days, the mycelium was inoculated into 200 × 1000 mL Erlenmeyer flasks, each containing 300 mL of liquid medium (20 g sorbitol, 20 g maltose, 10 g monosodium glutamate, 0.5 g KH_2_PO_4_, 0.3 g MgSO_4_·7H_2_O, 0.5 g tryptophan, 3 g yeast extract per liter of sea water pH 6.5). The medium was incubated at 28 °C under static conditions for 41 days.

### 3.3. Extraction and Purification

The mycelium and the aqueous layer of the whole culture (60 L) were separated through cheesecloth. The mycelium was soaked by acetone for three times, and the acetone phases were collected and then evaporated under vacuum until acetone was removed. The residue water layer and the whole aqueous layer were extracted with three volumes of ethyl acetate (EtOAc) for three times. The EtOAc layer was collected and evaporated to dry. A total of 51.4 g of crude extract was obtained. The EtOAc extract was subjected to a silica gel VLC column, eluting with petroleum, petroleum/CH_2_Cl_2_ (*v*/*v*, 1:1), CH_2_Cl_2_, a stepwise gradient of CH_2_Cl_2_/MeOH (*v*/*v*, 100:1, 75:1,50:1, 25:1, 10:1, 5:1, 2:1, 1:1), and MeOH to give 11 fractions. Fraction 4 (400 mg) was subjected to Sephadex LH-20 chromatography (50 × 1270 mm) to give two fractions (Fr. 4-1–Fr. 4-2). Fraction 4-2 was further purified by HPLC over an ODS column (80% MeOH−H_2_O, *v*/*v*) to give compounds **8** (*t*_R_ 16.6 min, 3.6 mg) and **9** (*t*_R_ 20.9 min, 6.3 mg) and a mixture (20 mg) of **7** and **10**. The mixture was then subjected to Sephadex LH-20 chromatography (30 × 80 mm) and further purified by HPLC over the ODS column (75% MeOH−H_2_O, *v*/*v*) to give compounds **7** (*t*_R_ 11.1 min, 12.0 mg) and **10** (*t*_R_ 26.8 min, 3.0 mg). Fraction 4-1 was subjected to Sephadex LH-20 chromatography (30 × 80 mm) to give Fr. 4-1-1–Fr. 4-1-2. Fraction 4-1-1 was then purified by HPLC over an ODS column (25% MeOH−H_2_O, *v*/*v*) to give compound **12** (*t*_R_ 15.2 min, 26.0 mg). Fraction 4-1-2 was further subjected to Sephadex LH-20 chromatography (20 × 78 mm) to give 4 fractions (Fr. 4-1-2-1–Fr. 4-1-2-4). Fraction 4-1-2-1 was purified by HPLC over an ODS column (70% MeOH−H_2_O, *v*/*v*) to give compounds **1** (*t*_R_ 35.4 min, 3.0 mg), **2** (*t*_R_ 42.2 min, 4.8 mg), and **3** (*t*_R_ 30.9 min, 3.0 mg). Fraction 4-1-2-2 was further subjected to a silica column, eluting with petroleum ether/EtOAc to give eight fractions (Fr. 4-1-2-2-1–Fr. 4-1-2-2-8). Fraction 4-1-2-2-1 was purified by HPLC over an ODS column (70% MeOH−H_2_O, *v*/*v*) to give compound **14** (*t*_R_ 24.0 min, 4.5 mg). Fraction 4-1-2-2-5 was purified by HPLC over an ODS column (75% MeOH−H_2_O, *v*/*v*) to give compound **13** (*t*_R_ 20.3 min, 3.0 mg). Fraction 4-1-2-2-8 was further subjected to a silica column, eluting with petroleum ether/EtOAc to give compound **11** (ODS, 75% MeOH−H_2_O, *v*/*v*, *t*_R_ 20.2 min, 8.0 mg). Fraction 7 was further subjected to a silica column, eluting with petroleum ether/EtOAc to give two fractions (Fr. 7-1 and Fr. 7-2). Fraction 7-1 was subjected to a silica column, eluting with petroleum ether/EtOAc and further purified by HPLC over an ODS column (75% MeOH−H_2_O, *v*/*v*) to give compound **15** (*t*_R_ 9.9 min, 100.0 mg). Fraction 7-2 was subjected to a silica LC column, eluting with petroleum ether/EtOAc to give Fr. 7-2-1 and Fr. 7-2-2. Fraction 7-2-1 was further purified by HPLC over an ODS column (75% MeOH−H_2_O, v/v) to give compound **4** (*t*_R_ 11.3 min, 8.6 mg). Fraction 7-2-2 was further purified by HPLC over an ODS column (75%MeOH−H_2_O, *v*/*v*) to give compounds **16** (*t*_R_ 4.2 min, 25.6 mg) and **5** (*t*_R_ 5.5 min, 45.9 mg). Fraction 8 was further applied to a silica LC column, eluting with petroleum ether/EtOAc, and further purified by HPLC over an ODS column (60% MeOH−H_2_O, *v*/*v*) to give compound **6** (*t*_R_ 4.39 min, 20.1 mg).

**Sclerotiorin A (1)**: yellow amorphous powder; ${\left[\alpha \right]}_{D}^{25}$ + 45.7 (*c* 0.2, EtOH); UV (MeOH) *λ*_max_ (log *ε*) 255 (2.07), 361 (2.10), 391 (2.12); ECD (0.6 mM, MeOH) λ_max_ (*Δε*) 233 (+0.52), 258 (−0.53), 317 (+2.59,) and 394 (–0.46) nm; IR (KBr) *ν*_max_ 3526, 1618, 1400 cm^−1^; ^1^H and ^13^C NMR data, see Table 1 and Table 2; HRESIMS *m*/*z* 405.1834 [M + H]^+^ (calcd. for C_23_H_30_O_4_Cl, 405.1827).

**Sclerotiorin B (2)**: yellow amorphous powder; ${\left[\alpha \right]}_{D}^{25}$ + 6.6 (*c* 0.1, EtOH); UV (MeOH) *λ*_max_ (log *ε*) 258 (1.67), 370 (1.76), 390 (1.79); ECD (0.6 mM, MeOH) *λ*_max_ (*Δε*) 228 (+0.45), 258 (−0.68), 317 (+2.45), and 387 (−0.10) nm; IR (KBr) *ν*_max_ 3599, 1622, 1513, 1372 cm^−1^; ^1^H and ^13^C NMR data, see Table 1 and Table 2; HRESIMS *m*/*z* 405.1836 [M + H]^+^ (calcd. for C_23_H_30_O_4_Cl, 405.1827).

**Sclerotiorin C (3)**: yellow amorphous powder; ${\left[\alpha \right]}_{D}^{25}$ + 10.7 (*c* 0.1, EtOH); UV (MeOH) *λ*_max_ (log *ε*) 248 (0.85), 367 (0.95), 391 (1.02); ECD (0.1 mM, MeOH) λ_max_ (*Δε*) 225 (+0.52), 253 (−0.14), 324 (+2.09), and 387 (–1.00) nm; IR (KBr) *ν*_max_ 3465, 1622, 1388 cm^−1^; ^1^H and ^13^C NMR data, see Table 1 and Table 2; HRESIMS *m*/*z* 371.2218 [M + H]^+^ (calcd. for C_23_H_31_O_4_, 371.2217).

**Sclerotiorin D (4)**: red powder; ${\left[\alpha \right]}_{D}^{25}$ + 207.5 (*c* 0.025, MeOH); UV (MeOH) *λ*_max_ (log *ε*) 236(2.46), 369 (2.56); ECD (0.5 mM, MeOH) λ_max_ (*Δε*) 244 (+3.7), 303 (−5.98), and 383 (+4.6) nm; IR (KBr) *ν*_max_ 2921, 2365, 1739, 1591, 1502, 1240 cm^−1^; ^1^H and ^13^C NMR data, see Table 1 and Table 2; HRESIMS *m*/*z* 490.1993 [M + H]^+^ (calcd. for C_26_H_33_O_6_NCl, 490.1991).

**Sclerotiorin E(5)**: red powder, ${\left[\alpha \right]}_{D}^{25}$ + 143.7 (*c* 0.05, MeOH); ECD (0.72 mM, MeOH) λ_max_ (Δ*ε*) 223 (−1.43), 244 (+6.68), 305 (−12.21), and 385 (+9.45) nm; IR (KBr) *ν*_max_ 3443, 2956, 2357, 1707, 1590, 1509, 1236 cm^−1^; ^1^H NMR (600 MHz, CDCl_3_) and ^13^C NMR (150 MHz, CDCl_3_) shown in Tables S1–S2; HRESIMS *m*/*z* 833.3350 [M + H]^+^ (calcd. for C_46_H_55_O_8_N_2_Cl_2_, 833.3330).

### 3.4. Conversion from ***7*** to ***14***

To a solution of compound **7** (15 mg) in 2 ml of THF, 300 mg of NH_4_OAc and 300 μL of MeOH were added. The mixture was stirred at 25 °C for 17 h. The reaction mixture was diluted with 5 mL of water and extracted three times with equal volumes of EtOAc. The EtOAc layers were combined, washed with water, dried over anhydrous Na_2_SO_4_, filtered, and concentrated in vacuo to yield a product as an orange oil. The orange oil was purified by semipreparative HPLC on ODS (70% acetonitrile/H_2_O, *v*/*v*) to afford compound **14** (6.0 mg, *t*_R_ 5.6 min, 40% yield) that was identified by the same retention time in the co-HPLC (Figure S37) and the same sign of specific rotation as natural **14**.

### 3.5. Conversion from ***7*** to ***15***

Amino ethanol (20 μL) was added to 1 mL of CH_2_Cl_2_ solution of compound **7** (6 mg). The reaction solution was stirred at 25 °C for 24 h under a nitrogen atmosphere. After CH_2_Cl_2_ was dried in vacuo, the mixture was purified by semipreparative HPLC on an ODS column (85% acetonitrile/H_2_O, *v*/*v*) to afford compound **15** (6.1 mg, *t*_R_ 5.6 min, 92% yield), which was identified by the same retention time in the co-HPLC (Figure S37) and the same sign of specific rotation as natural **15**.

### 3.6. Conversion from ***7*** to ***16***

Amino butyric acid (21.5 mg) was added to 2 mL of DMF solution of compound **7** (8 mg). The solution was stirred at 25 °C for 5 h under a nitrogen atmosphere, and then 10 mL of H_2_O was added. The reaction mixture was extracted three times with equal volumes of EtOAc. The EtOAc layers were combined, dried over anhydrous Na_2_SO_4_, filtered, and concentrated in vacuo to yield an orange oil. The orange oil was purified by semipreparative HPLC on an ODS column (85% acetonitrile/H_2_O, *v*/*v*) to afford compound **16** (9.0 mg, *t*_R_ 7.9 min, 93% yield), which was identified by the same retention time in the co-HPLC (Figure S37) and the same sign of specific rotation as natural **16**. 

### 3.7. Conversion from ***14*** to ***5***

1,4-Diiodobutane (6.96 μL) was added to the acetone solution (3 mL) of compound **14** (20 mg) and K_2_CO_3_ (21.2 mg). The reaction solution was heated to 50 °C and stirred for 7 days under a nitrogen atmosphere. The reaction solution was concentrated in vacuo and dissolved in 10 mL of H_2_O. The obtained solution was extracted three times with equal volumes of EtOAc. The EtOAc layers were combined, dried over anhydrous Na_2_SO_4_, filtered, and concentrated in vacuo to give a gum that was purified by semipreparative HPLC on an ODS column (85% acetonitrile/H_2_O, *v*/*v*) to afford compound **5** (1.41 mg, *t*_R_ 7.9 min, 3.3% yield), which was identified by the same retention time in the co-HPLC (Figure S37) and the same sign of specific rotation as natural **5**.

### 3.8. Conversion from ***16*** to ***4***

A volume of 10 μL of CH_3_I was added to the acetone solution of compound **16** (1.33 mg) and K_2_CO_3_ (5.50 mg). The solution was stirred at 28 °C for 24 h under a nitrogen atmosphere, and then 10 mL of H_2_O was added. The mixture was extracted three times with equal volumes of CH_2_Cl_2_. After CH_2_Cl_2_ was dried in vacuo, the CH_2_Cl_2_ extracts were applied to semipreparative HPLC on an ODS column (80% acetonitrile/H_2_O, *v*/*v*) to afford compound **4** (1.28 mg, *t*_R_ 5.40 min, 94% yield), which was identified by the same retention time in the co-HPLC (Figure S37) and the same sign of specific rotation as natural **4**.

### 3.9. X-ray Crystal Data for ***14*** (Mo Kα Radiation)

Red orthorhombic crystal from MeOH; molecular formula C_21_H_25_O_4_NCl; space group P2_1_ with *a* = 8.6912 (7) Å, *b* = 7.4026 (6) Å, *c* = 16.0515 (14) Å, *V* = 996.39 (14) Å^3^, absolute structure Flack parameter: 0.01(2). *Z* = 2, D*_calcd_* = 1.299 mg/m^3^, *μ* = 1.913 mm^−1^, and *F* (000) = 412; crystal size 0.35 × 0.12 × 0.07 mm^3^. T = 293(2) K. A total of 5671 unique reflections (2*θ* < 140°) were collected on a Bruker Smart CCD area detector diffractometer with graphite monochromated Mo Kα radiation (*λ* = 1.54178 Å). The structure was solved by direct methods (SHELXS-97) and expanded using Fourier techniques (SHELXL-97). The final cycle of full-matrix least-squares refinement was based on 2612 unique reflections (2*θ* < 140°) and 249 variable parameters and converged with unweighted and weighted agreement factors of *R*_1_ = 0.0426 and *wR*_2_ = 0.0952 for *I* > 2*σ* (*I*) data. Crystallographic data for **14** have been deposited in the Cambridge Crystallographic Data Centre as supplementary publication no. CCDC 1055936. Copies of the data can be obtained, free of charge, on application to CCDC, 12 Union Road, Cambridge CB2 1EZ, U.K. (Fax: +44 (0)-1223-336033; e-mail: deposit@ccdc.cam.ac.uk).

### 3.10. X-ray Crystal Data for ***15*** (Mo Kα Radiation)

Red orthorhombic crystal from MeOH; molecular formula C_23_H_29_O_5_NCl; space group *P*2_1_2_1_2_1_ with *a* = 6.7149(3) Å, *b* = 8.5574 (5) Å, *c* = 40.085 (2) Å, *V* = 2303.4 (2) Å^3^, absolute structure Flack parameter: −0.2(2). *Z* = 4, D*_calcd_* = 1.251 mg/m^3^, *μ* = 0.774 mm^−1^, and *F* (000) = 920; crystal size 0.40 × 0.11 × 0.08 mm^3^. T = 298(2) K. A total of 10,946 unique reflections (2*θ* < 50°) were collected on a Bruker Smart CCD area detector diffractometer with graphite monochromated Mo Kα radiation (*λ* = 0.71073 Å). The structure was solved by direct methods (SHELXS-97) and expanded using Fourier techniques (SHELXL-97). The final cycle of full-matrix least-squares refinement was based on 3965 unique reflections (2*θ* < 50°) and 288 variable parameters and converged with unweighted and weighted agreement factors of *R*_1_ = 0.1023 and *wR*_2_ = 0.2473 for *I* > 2*σ*(*I*) data. Crystallographic data for **15** have been deposited in the Cambridge Crystallographic Data Centre as supplementary publication no. CCDC 1056013. Copies of the data can be obtained, free of charge, on application to CCDC, 12 Union Road, Cambridge CB2 1EZ, U.K. (Fax: +44 (0)-1223-336033; e-mail: deposit@ccdc.cam.ac.uk)

### 3.11. Anti-influenza A (H1N1) Virus Bioassay

The antiviral activity against H1N1 was examined by the CPE+MTT assay [38,39]. MDCK cells were cultured in RPMI-1640 medium containing 10% fetal bovine serum in 5% CO_2_ at 37 °C. When the cells reached 70–80% confluency, they trypsinized into individual cells, and the cell suspension concentration was adjusted to 1 × 10^5^ cells/mL. The cells were seeded in a 96-well plate in 100 μL per well and cultured at 37 °C, 5% CO_2,_ for 18 h. After eliminating the culture medium, the influenza virus (A/Puerto Rico/8/34 (H1N1), PR/8), diluted to 100 TCID_50_, was added (100 μL per well) to the 96-well plate; an equal amount of virus-free dilution was used as a negative control. The 96-well plate was incubated for 1 h at 37 °C, 5% CO_2_. The samples to be tested and a positive-control drug were diluted in PBS buffer, and 20 μL of these solutions was added into the wells. PBS buffer was used as a negative control. After incubation for 48 h at 37 °C, the cells were fixed with 100 μL of 4% formaldehyde for 20 min at room temperature, then the formaldehyde was poured out, and 50 μL of 0.1% crystal violet stain was added, staining for 30 min at 37 °C. After the plates were washed and dried, the absorbance (OD) of each well was measured at 570 nm with a microplate reader (Bio-Rad, USA). The IC_50_, as the compound concentration required to inhibit influenza virus yield at 48 h post-infection by 50%, was calculated. The IC_50_ value of the positive control, Ribavirin, was 179.8 μM.

### 3.12. Anti-α-glycosidase Bioassay

The PNPG method [40] used to evaluate the inhibitory activity against α-glycosidase was described in our previous work [41].

## 4. Conclusions

Four new azaphilones, sclerotiorins A–D (**1**–**4**), along with 12 known analogues (**5**–**16**) were isolated and identified from a fermentation broth of the sponge-derived fungus, *P. sclerotiorum* OUCMDZ-3839. Compounds **5**, **7**, **10**, **12**–**14,** and **16** showed stronger antiviral activity against H1N1 in the MDCK cell line than the positive control Ribavirin. Additionally, compounds **11** and **14** displayed significant inhibitory activity against *α*-glycosidase, with IC_50_ values of 17.3 and 166.1 μM, respectively.

## Figures and Tables

**Table 1:** ^13^C (125 MHz) NMR data for compounds **1**–**4** (*δ* in ppm).

Position	1 ^a^	2 ^a^	3 ^a^	4 ^b^
	*δ*_C_	*δ*_C_	*δ*_C_	*δ*_C_
1	144.6, CH	145.4, CH	145.4, CH	141.2, CH
3	157.4, C	157.7, C	155.4, C	148.3, C
4	105.0, CH	104.9, CH	107.7, CH	111.7, CH
4a	138.9, C	139.6, C	143.0, C	144.9, C
5	109.0, C	108.4, C	106.1, CH	102.3, C
6	187.7, C	187.9, C	195.1, C	184.5, C
7	83.5, C	84.1, C	83.1, C	84.8, C
8	43.4, CH	42.7, CH	43.2, CH	193.9, C
8a	116.4, C	115.7, C	116.4, C	115.1, C
9	117.6, CH	117.5, CH	117.4, CH	114.6, CH
10	140.3, CH	140.5, CH	139.0, CH	145.6, CH
11	132.2, C	132.2, C	132.0, C	132.2, C
12	146.2, CH	146.4, CH	145.3, CH	148.4, CH
13	34.3, CH	34.3, CH	34.2, CH	35.2, CH
14	29.6, CH_2_	29.6, CH_2_	29.6, CH_2_	30.0, CH_2_
15	11.8, CH_3_	11.8, CH_3_	11.9, CH_3_	12.1, CH_3_
16	20.2, CH_3_	20.2, CH_3_	20.3, CH_3_	20.3, CH_3_
17	12.3, CH_3_	12.3, CH_3_	12.3, CH_3_	12.6, CH_3_
18	23.9, CH_3_	24.4, CH_3_	24.3, CH_3_	23.4, CH_3_
19	106.0, C	105.4, C	105.1, C	170.3, C
20	45.8, CH_2_	44.2, CH_2_	44.4, CH_2_	20.4, CH_3_
21	22.9, CH_3_	22.8, CH_3_	23.0, CH_3_	53.4, CH_2_
22	-	-	-	25.4, CH_2_
23	-	-	-	30.2, CH_2_
24	-	-	-	172.6, C
OCH_3_	48.3, CH_3_	48.3, CH_3_	48.3, CH_3_	52.2, CH_3_

^a^ recorded in DMSO-*d*_6_. ^b^ recorded in CDCl_3_.

**Table 2:** ^1^H (500 MHz) NMR data for compounds **1**–**4** (*δ* in ppm).

Position	1 ^a^	2 ^a^	3^a^	4 ^b^
	*δ*_H_ (*J* in Hz)	*δ*_H_ (*J* in Hz)	*δ*_H_ (*J* in Hz)	*δ*_H_ (*J* in Hz)
1	7.65, s	7.80, s	7.65, s	7.76, s
4	6.67, s	6.69, s	6.40, s	7.05, s
5	-	-	5.23, s	-
8	3.23, dd (10.3, 10.3)	3.42, dd (12.7, 10.3)	3.30, m	-
9	6.44, d (15.8)	6.45, d (15.8)	6.18, d (15.8)	6.31, d (15.3)
10	6.99, d (15.8)	7.01, d (15.8)	6.90, d (15.8)	6.98, d (15.3)
12	5.71, d (9.7)	5.72, d (9.6)	5.65, d (9.7)	5.71, d (9.7)
13	2.46, m	2.46, m	2.45, m	2.48, m
14	1.39, m; 1.26, m	1.39, m; 1.27, m	1.37, m; 1.27, m	1.44, m; 1.36, m
15	0.81, t (7.5)	0.81, t (7.5)	0.81, t (7.4)	0.88, t (7.4)
16	0.96, d (6.6)	0.96, d (6.6)	0.95, d (6.6)	1.02, d (6.6)
17	1.80, s	1.80, s	1.77, s	1.90, s
18	1.19, s	1.25, s	1.20, s	1.55, s
20	2.36, dd (13.0,10.0) 1.89, dd (13.0,10.5)	2.18, dd (12.7,7.3) 1.87, dd (12.7,10.5)	2.15, dd (13.1,10.0) 1.84, m	2.16, s
21	1.35, s	1.25, s	1.23, s	3.95, t (7.8)
22	-	-	-	2.05, m
23	-	-	-	2.43, t (6.4)
OCH_3_	3.03, s	3.20, s	3.19, s	3.70, s

^a^ recorded in DMSO-*d*_6_. ^b^ recorded in CDCl_3_.

**Table 3:** Activity against H1N1 of compounds **5**, **7**, **10**, **12**–**14,** and **16**. IC_50_.

Compound	5	7	10	12	13	14	16	Ribavirin
IC_50_ (μM)	78.6	128.7	115.0	150.5	91.4	133.9	156.8	179.8

## Supplementary Materials

The following are available online at https://www.mdpi.com/1660-3397/17/5/260/s1, The ITS rRNA sequences data of *Penicillium* sp. OUCMDZ-3839; Tables S1–S3: NMR data of known compounds (**5**–**16**); physicochemical data of known compounds **5**–**16**; specific rotation of synthetic compounds **4**, **5** and **14**–**16**; Figure S1: HRESIM Spectrum of Compound **1**; Figures S2–S9: NMR Spectrum of Compound **1** in DMSO-*d_6_*; Figure S10: HRESIM Spectrum of Compound **2**; Figures S11–S17: NMR Spectrum of Compound **2** in DMSO-*d_6_*; Figure S18: HRESIM Spectrum of Compound **3**; Figures S19–S25: NMR Spectrum of Compound **3** in DMSO-*d_6_*; Figure S26: HRESIM Spectrum of Compound **4**; Figures S27–S32: NMR Spectrum of Compound **4** in CDCl_3_; Figure S33: HRESIM Spectrum of Compound **5**; Figures S34–S36: NMR Spectrum of Compound **5** in CDCl_3_; Figure S37: Co-HPLC profiles of the synthetic and the natural **4**, **5** and **14**–**16**; Figure S38: Measured ECD curves of compounds **4**, **5**, **7** and **14**–**16**.
