# Supplementary material for: Azaphilones from the Marine Sponge-Derived Fungus Penicillium sclerotiorum OUCMDZ-3839
Source: Mar Drugs. 2019 Apr 30;17(5):260. doi: 10.3390/md17050260 (PMC6563140; doi:10.3390/md17050260)
Supplement: Supplementary file 1 [file marinedrugs-17-00260-s001.zip › supplementary material/marinedrugs-489570_SI_R1 (marked).docx]

**Supporting Information**

**Azaphilones from the Marine** **Sponge-Derived Fungus *Penicillium sclerotiorum* OUCMDZ-****3839**

Qian Jia^1,2,†^, Yuqi Du^1,2,†^, Chen Wang^1^, Yi Wang^1^, Tonghan Zhu^1,3^ and Weiming Zhu^1,2,^*

^1^ Key Laboratory of Marine Drugs, Ministry of Education of China, School of Medicine and Pharmacy, Ocean University of China, Qingdao 266003, P. R. China

^2^ Open Studio for Druggability Research of Marine Natural Products, Laboratory for Marine Drugs and Bioproducts, Pilot National Laboratory for Marine Science and Technology (Qingdao), Qingdao 266003, China

^3^ College of Computer Science and Engineering, Shandong University of Science and Technology, Qingdao 266590, China

^*^ Correspondence: weimingzhu@ouc.edu.cn (W.Z.); Tel./Fax: +86-532-8203-1268 (W.Z.)

^†^ These authors contributed equally to this paper.

**List of Supporting Information**

The ITS rRNA Gene Sequences Data for *P*. *sclerotiorum* OUCMDZ-3839S4

**Table S1**. The ^13^C-NMR Data of Known Compounds **5**−**16**…….S5

**Table S2**. The ^1^H-NMR Data of Known Compounds **5**−**10**…….S6

**Table S3**. The ^1^H-NMR Data of Known Compounds **11**−**16**……S7

The Physicochemical Data of Known Compounds **6−16**S8

The Specific Rotation of Synthetic Compounds **4**, **5 and 14−16**S9

**Figure S1**. The HRESIM Spectrum of Compound **1**S10

**Figure S2**. The ^1^H-NMR Spectrum of Compound **1** in DMSO-*d_6_*S11

**Figure S3**. The ^13^C-NMR Spectrum of Compound **1** in DMSO-*d_6_*S12

**Figure S4**. The DEPT Spectrum of Compound **1** in DMSO-*d_6_*S13

**Figure S5**. The HSQC Spectrum of Compound **1** in DMSO-*d_6_*S14

**Figure S6**. The ^1^H-^1^H COSY Spectrum of Compound **1** in DMSO-*d_6_*………..S16

**Figure S7**. The HMBC Spectrum of Compound **1** in DMSO-*d_6_*S19

**Figure S8**. The NOESY Spectrum of Compound **1** in DMSO-*d_6_*S24

**Figure S9**. The NOE Difference Spectrum of Compound **1** in DMSO-*d_6_*S26

**Figure S10**. The HRESIM Spectrum of Compound **2**S27

**Figure S11**. The ^1^H-NMR Spectrum of Compound **2** in DMSO-*d_6_*S28

**Figure S12**. The ^13^C-NMR Spectrum of Compound **2** in DMSO-*d_6_*S29

**Figure S13**. The DEPT Spectrum of Compound **2** in DMSO-*d_6_*S30

**Figure S14**. The HSQC Spectrum of Compound **2** in DMSO-*d_6_*S31

**Figure S15**. The ^1^H-^1^H COSY Spectrum of Compound **2** in DMSO-*d_6_*S33

**Figure S16**. The HMBC Spectrum of Compound **2** in DMSO-*d_6_*S36

**Figure S17**. The NOESY Spectrum of Compound **2** in DMSO-*d_6_*S41

**Figure S18**. The HRESIM Spectrum of Compound **3**S42

**Figure S19**. The ^1^H-NMR Spectrum of Compound **3** in DMSO-*d_6_*S43

**Figure S20**. The ^13^C-NMR Spectrum of Compound **3** in DMSO-*d_6_*S44

**Figure S21**. The DEPT Spectrum of Compound **3** in DMSO-*d_6_*S45

**Figure S22**. The HSQC Spectrum of Compound **3** in DMSO-*d_6_*S46

**Figure S23**. The ^1^H-^1^H COSY Spectrum of Compound **3** in DMSO-*d_6_*S48

**Figure S24**. The HMBC Spectrum of Compound **3** in DMSO-*d_6_*S51

**Figure S25**. The NOESY Spectrum of Compound **3** in DMSO-*d_6_*S56

**Figure S26**. The HRESIM Spectrum of Compound **4**S57

**Figure S27**. The ^1^H-NMR Spectrum of Compound **4** in CDCl_3_S58

**Figure S28**. The ^13^C-NMR Spectrum of Compound **4** in CDCl_3_S59

**Figure S29**. The DEPT Spectrum of Compound **4** in CDCl_3_S60

**Figure S30**. The HSQC Spectrum of Compound **4** in CDCl_3_S61

**Figure S31**. The ^1^H-^1^H COSY Spectrum of Compound **4** in CDCl_3_S63

**Figure S32**. The HMBC Spectrum of Compound **4** in CDCl_3_S66

**Figure S33**. The HRESIM Spectrum of Compound **5**S72

**Figure S34**. The ^1^H-NMR Spectrum of Compound **5** in CDCl_3_S73

**Figure S35**. The ^13^C-NMR Spectrum of Compound **5** in CDCl_3_S74

**Figure S36**. The DEPT Spectrum of Compound **5** in CDCl_3_S75

**Figure S37**. Co-HPLC profiles of the synthetic and the natural **4**, **5** and **14**−**16**…S76

**Figure S38**. Measured ECD curves of compounds **4**, **5**, **7** and **14**−**16**S77

**Figure S39**. HPLC of the reaction products from **14** to **5**S78

**The ITS rRNA Gene Sequences Data for *P*. *sclerotiorum* OUCMDZ-3839**

GCGGAAGGATCATTACTGAGTGAGGGCCCTCTGGGTCCAACCTCCCACCCGTGTTTATTGTACCTTGTTGCTTCGGCAGGCCCGCCTCACGGCCGCCGGGGGGCTTTCCGCCCCCGGGCCCGCGCCTGCCGGAGACAATCTTGAACGCTGTCTGAAGAATGCAGTCTGAGCGATTAAGCAAAATTAGTTAAAACTTTCAACAACGGATCTCTTGGTTCCGGCATCGATGAAGAACGCAGCGAAATGCGATAATTAATGTGAATTGCAGAATTCAGTGAATCATCGAGTCTTTGAACGCACATTGCGCCCCCTGGTATTCCGGGGGGCATGCCTGTCCGAGCGTCATTGCTGCCCTCAAGCCCGGCTTGTGTGTTGGGCCCTGTTCCCCCGGGAACAGGCCCGAAAGGCAGTGGCGGCACCGCGTCCGATCCTCGAGCGTATGGGGCTTTGTCACCCGCTCTGTAGGCCCGGCCGGCGCTTGCCCCCCATCAATCTTTTTTTCAGGTTGACCTCGGATCAGGTAGGGATACCCGCTGAACTTAAGCAT

**Table S1**.^13^C (125MHz) NMR Data for Compounds **5-16**

| position | **5** ^a^ | **6** ^b^ | **7**^a^ | **8** ^a^ | **9** ^a^ | **10** ^a^ | **11** ^a^ | **12** ^a^ | **13** ^b^ | **14** ^a^ | **15** ^a^ | **16** ^a^ |
| --- | --- | --- | --- | --- | --- | --- | --- | --- | --- | --- | --- | --- |
| 1 | 141.5, CH | 68.5, CH_2_ | 152.7, CH | 67.5, CH_2_ | 67.3, CH_2_ | 147.9, CH | 147.3, CH | 146.6, CH | 67.99, CH_2_ | 138.9, CH | 142.7, CH | 141.5, CH |
| 3 | 148.5, C | 162.1, C | 158.2, C | 163.0, C | 162.3, C | 158.1, C | 157.5, C | 159.0, C | 161.6, C | 146.9, C | 145.3, C | 148.6, C |
| 4 | 111.8, CH | 100.2, CH | 106.5, CH | 102.4, CH | 101.3, CH | 105.5, CH | 107.7, CH | 105.2, CH | 104.2, CH | 110.7, CH | 111.7, CH | 111.5, CH |
| 4a | 144.3, C | 145.8, C | 138.8, C | 145.3, C | 144.9, C | 140.4, C | 145.3, C | 141.0, C | 151.6, C | 147.98, C | 149.4, C | 145.4, C |
| 5 | 102.1, C | 118.2, C | 114.7, C | 116.5, C | 118.1, C | 106.3, C | 106.0, CH | 109.2, C | 115.7, CH | 101.1, C | 100.9, C | 101.8, C |
| 6 | 183.8, C | 189.5, C | 191.9, C | 192.2, C | 187.4, C | 190.9, C | 191.4, C | 184.2, C | 194.4, C | 183.9, C | 184.1, C | 184.4, C |
| 7 | 85.7, C | 75.3, C | 84.7, C | 76.1, C | 74.4, C | 74.5, C | 82.9, C | 83.5, C | 74.6, C | 85.2, C | 85.0, C | 84.9, C |
| 8 | 194.3, C | 72.6, CH | 186.1, C | 74.3, CH | 73.1, CH | 72.6, CH | 42.9, CH | 42.5, CH | 74.3, CH | 193.4, C | 193.8, C | 193.9, C |
| 8a | 114.7, C | 37.0, CH | 110.9, C | 36.9, CH | 35.5, CH | 115.4, C | 114.2, C | 113.4, C | 35.1, CH | 114.6, C | 114.7, C | 115.1, C |
| 9 | 114.3, CH | 120.4, CH | 115.8, C | 119.3, CH | 119.8, CH | 116.3, CH | 116.1, CH | 116.1, CH | 118.9, CH | 116.7, CH | 115.3, C | 114.6, CH |
| 10 | 146.2, CH | 144.8, CH | 143.0, CH | 142.9, CH | 140.4, CH | 142.3, CH | 141.9, CH | 143.0, CH | 140.8, CH | 143.3, CH | 145.9, CH | 145.8, CH |
| 11 | 132.2, C | 75.0, C | 132.1, C | 132.9, C | 132.3, C | 132.0, C | 131.9, C | 132.0, C | 132.3, C | 132.1, C | 131.9, C | 132.3, C |
| 12 | 148.3, CH | 79.3, CH | 149.0, CH | 148.2, CH | 145.9, CH | 148.2, CH | 147.8, CH | 148.7, CH | 146.4, CH | 148.8, CH | 148.0, CH | 148.5, CH |
| 13 | 35.2, CH | 34.5, CH | 35.3, CH | 35.1, CH | 34.3, CH | 35.2, CH | 35.1, CH | 35.2, CH | 34.8, CH | 35.2, CH | 35.0, CH | 35.2, CH |
| 14 | 30.1, CH_2_ | 28.6, CH_2_ | 30.2, CH_2_ | 30.2, CH_2_ | 29.6, CH_2_ | 30.2, CH_2_ | 30.2, CH_2_ | 30.2, CH_2_ | 30.3, CH_2_ | 30.2, CH_2_ | 30.0, CH_2_ | 30.1, CH_2_ |
| 15 | 12.1, CH_3_ | 11.9, CH_3_ | 12.1, CH_3_ | 12.1, CH_3_ | 11.9, CH_3_ | 12.1, CH_3_ | 12.1, CH_3_ | 12.1, CH_3_ | 12.1, CH_3_ | 12.1, CH_3_ | 12.0, CH_3_ | 12.1, CH_3_ |
| 16 | 20.2, CH_3_ | 14.1, CH_3_ | 20.3, CH_3_ | 20.4, CH_3_ | 20.2, CH_3_ | 20.3, CH_3_ | 20.6, CH_3_ | 20.3, CH_3_ | 20.5, CH_3_ | 20.3, CH_3_ | 20.2, CH_3_ | 20.3, CH_3_ |
| 17 | 12.6, CH_3_ | 27.2, CH_3_ | 12.5, CH_3_ | 12.5, CH_3_ | 12.3, CH_3_ | 12.5, CH_3_ | 12.5, CH_3_ | 12.2, CH_3_ | 12.5, CH_3_ | 12.5, CH_3_ | 12.6, CH_3_ | 12.6, CH_3_ |
| 18 | 23.6, CH_3_ | 19.8, CH_3_ | 22.7, CH_3_ | 24.7, CH_3_ | 19.6, CH_3_ | 21.3, CH_3_ | 23.5, CH_3_ | 23.5, CH_3_ | 20.2, CH_3_ | 23.8, CH_3_ | 23.3, CH_3_ | 23.4, CH_3_ |
| 19 | 170.2, C |  | 170.2, C | 170.8, C | 170.4, C | 170.8, C | 168.6, C | 168.2, C | 170.6, C | 171.1, C | 170.2, C | 170.4, C |
| 20 | 20.5, CH_3_ |  | 20.2, CH_3_ | 20.8, CH_3_ | 20.7, CH_3_ | 24.5, CH_3_ | 57.4, CH | 57.3, CH | 20.8, CH_3_ | 20.6, CH_3_ | 20.3, CH_3_ | 20.4, CH_3_ |
| α /β-OCH_3_ |  |  |  |  | 48.3, CH_3_ |  |  |  |  |  |  |  |
| 21 | 53.2, CH_2_ |  |  |  | 23.0, CH_3_ |  | 200.1, C | 199.8, C |  |  | 56.1, CH_2_ | 53.7, CH_2_ |
| 22 | 27.1, CH_2_ |  |  |  |  |  | 30.3, CH_3_ | 30.3, CH_3_ |  |  | 60.5, CH_2_ | 25.2, CH_2_ |
| 23 |  |  |  |  |  |  |  |  |  |  |  | 30.2, CH_2_ |
| 24 |  |  |  |  |  |  |  |  |  |  |  | 175.0, C |

a *δ*= ppm in CDCl_3_

b *δ*= ppm in DMSO-*d*_6_

**Table S2**.^1^H (500 MHz) NMR Data for Compounds **5-10**

| position | **5** ^a^ | **6** ^b^ | **7** ^a^ | **8** ^a^ | **9** ^a^ | **10** ^a^ |
| --- | --- | --- | --- | --- | --- | --- |
|  | *δ*_H_ (*J* in Hz) | *δ*_H_ (*J* in Hz) | *δ*_H_ (*J* in Hz) | *δ*_H_ (*J* in Hz) | *δ*_H_ (*J* in Hz)*δ*_C_ | *δ*_H_ (*J* in Hz) |
| 1 | 8.07, s | 4.67, dd (10.9, 5.0)  3.83, dd (10.9, 13.7) | 7.92, s | 4.48, dd (4.8,10.8)  3.81, dd (12.9,10.8) | 4.47, dd (5.0,10.9)  3.79, dd (13.5,10.9) | 7.49, s |
| 4 | 6.95, s | 5.98, s | 6.64, s | 6.08, s | 6.15, s | 6.50, s |
| 7-OH |  |  |  | 5.63, brs | 5.97, brs |  |
| 8 |  | 3.35, ovl ^c^ |  | 5.52, d (2.9) | 4.91, d (10.0) | 5.57, s |
| 8a |  | 3.04, ddd (5.0, 9.4, 14.1) |  | 3.23, ddd (2.9, 4.8, 12.9) | 3.45, ddd (4.2,9.3,14.3) |  |
| 9 | 6.14, d (15.2) | 6.23, d (15.8) |  | 5.98, d (15.5) | 6.27, d (15.7) | 6.04, d (15.8) |
| 10 | 6.87, d (15.2) | 6.70, d (15.8) | 7.05, d (15.7) | 6.98, d (15.5) | 6.89, d (15.7) | 7.03, d (15.8) |
| 12 | 5.68, d (9.6) | 3.27, m | 5.69, d (9.7) | 5.62, d (9.8) | 5.64, d (9.6) | 5.66, d (9.7) |
| 13 |  | 1.67, m | 2.47, m | 2.45, m | 2.45, m | 2.47, m |
| 14 | 1.44, m; 1.36, m | 1.32, m; 1.18, m | 1.43, m; 1.31, m | 1.41, m; 1.29, m | 1.38, m; 1.25, m | 1.30, m; 1.40, m |
| 15 | 0.91, t (7.4) | 0.82, t (7.4) | 0.86, t (6.8) | 0.84, t (7.4) | 0.80, t (7.3) | 0.86, t (7.3) |
| 16 | 1.00, d (6.6) | 0.71, d (6.8) | 1.00, d (5.4) | 0.99, d (6.6) | 0.95, d (6.6) | 1.00, d (6.5) |
| 17 | 1.76, s | 1.23, s | 1.84, s | 1.80, s | 1.81, s | 1.83, s |
| 18 | 1.53, s | 1.28, s | 1.56, s | 1.45, s | 1.42, s | 1.35, s |
| 20 | 2.14, s |  | 2.16, s | 2.02 s | 2.22, s | 2.02, s |
| 21 | 3.87, m  3.77, m |  |  |  |  |  |
| 22 | 1.67, m |  |  |  |  |  |
| 12-OH |  | 4.67, m |  |  |  |  |
| 11-OH |  | 4.73, s |  |  |  |  |
| 8-OH |  | 5.30, d (8.4) |  |  |  |  |
| 7-OH |  | 5.53, s |  |  |  |  |

a: *δ*= ppm in CDCl_3_

b: *δ*= ppm in DMSO-*d*_6_

c: overlapped with other signals.

**Table S3**.^1^H (500 MHz) NMR Data for Compounds **11-16**

| position | **11**^a^ | **12** ^a^ | **13** ^b^ | **14** ^a^ | **15** ^a^ | **16** ^a^ |
| --- | --- | --- | --- | --- | --- | --- |
|  | *δ*_H_ (*J* in Hz) | *δ*_H_ (*J* in Hz) | *δ*_H_ (*J* in Hz) | *δ*_H_ (*J* in Hz) | *δ*_H_ (*J* in Hz) | *δ*_H_ (*J* in Hz) |
| 1 | 7.45, s | 7.42, s | 4.35, dd (5.1,10.5); 3.79, dd (12.7,12.7) | 8.06, s | 7.93, s | 7.82, s |
| 4 | 6.55, s | 6.10, s | 5.67, s | 6.97, s | 6.99, s | 7.04, s |
| 5 |  | 5.44, s | 5.76, s |  |  |  |
| 8 | 3.87, d (12.1) | 3.83, d (11.7) | 4.99, d (10.0) |  |  |  |
| 8a |  |  | 3.34, m |  |  |  |
| 9 | 6.06, d (15.7) | 5.94, d (15.7) | 5.89, d (15.5) | 6.23, d (16.1) | 6.28, d (15.3) | 6.32, d (15.3) |
| 10 | 7.04, d (15.7) | 6.97, d (15.7) | 6.89, d (15.5) | 7.17, d (16.1) | 6.89, d (15.3) | 6.98, d (15.3) |
| 12 | 5.67, d (9.7) | 5.62, d (9.3) | 5.55, d (9.6) | 5.73, d (9.7) | 5.65, d (9.4) | 5.70, d (9.6) |
| 13 | 2.67, m | 2.59, m | 2.44, m | 2.47, m | 2.43, m | 2.47, m |
| 14 | 1.41, m; 1.31, m | 1.41, m; 1.29, m | 1.40, m; 1.28, m | 1.30, m; 1.41, m | 1.29, m; 1.37, m | 1.43, m; 1.34, m |
| 15 | 0.86, t (7.2) | 0.85, t (7.0) | 0.84, t (7.4) | 0.85, t (7.3) | 0.83, t (6.0) | 0.87, t (7.4) |
| 16 | 1.00, d (6.5) | 0.99, d (6.6) | 0.98, d（6.5） | 1.00, d (6.6) | 0.97, d (6.5) | 1.00, d (6.6) |
| 17 | 1.83, s | 1.80, s | 1.78, s | 1.85, s | 1.80, s | 1.87, s |
| 18 | 1.61, s | 1.59, s | 1.35, s | 1.59, s | 1.45, s | 1.53, s |
| 20 | 3.82, d (12.2) | 3.78, d (12.0) | 2.22, s | 2.15, s | 2.06, s | 2.14, s |
| 21 |  |  |  |  | 4.01, m | 3.96, m |
| 22 | 2.47, s | 2.47, s |  |  | 3.82, s | 2.03, m |

a *δ*= ppm in CDCl_3_

b *δ*= ppm in DMSO-*d*_6_

**The Physicochemical Data of Known Compounds 6−16**

**Geumsanol G (6)**: yellow powder, [α]^25^_D_ −80.3 (*c* 0.3, MeOH ); ECD (*c* 1.20 mM, MeOH) λ_max_ (Δ*ε*) 231 (−0.86), 254 (+1.77), 280 (−0.49), 299 (+ 0.41) and 376 (−2.10) nm; ^1^H NMR (500 MHz, DMSO-*d*_6_) and ^13^C NMR (125 MHz, DMSO-*d*_6_) shown in TabS1–S2; ESIMS *m/z* 387.3 [M + H]^+^.

**(+)Sclerotiorin (7)**: yellow powder, [α]^25^_D_ +198.1 (*c* 0.3, EtOH); ECD (*c* 1.28 mM, MeOH) λ_max_ (Δ*ε*) 218 (−3.26), 287 (+1.30), 340 (−0.85) and 391 (+2.87)nm; ^1^H NMR (500 MHz, CDCl_3_) and ^13^C NMR (125 MHz, CDCl_3_) shown in TabS1–S2; ESIMS *m/z* 413.2 [M + Na]^+^.

**Isochromopilone VIII (8)**: yellow powder, [α]^25^_D_ +120.8 (*c* 0.7, MeOH); ECD (*c* 1.20 mM, MeOH) λ_max_ (Δ*ε*) 233 (+0.7), 280 (−0.06), 319 (−0.42) and 370 (+1.02) nm;^1^H NMR (500 MHz, CDCl_3_) and ^13^C NMR (125 MHz, CDCl_3_) shown in Table S1–S2; ESIMS *m/z* 417.2 [M + Na]^+^.

**Isochromopilone IV (9)**: yellow powder, [α]^25^_D_ −91.3 (*c* 0.4, MeOH); ECD (*c* 1.0 mM, MeOH) λ_max_ (Δ*ε*) 208 (+5.32), 248 (−2.02), 279 (+2.76) and 397 (−6.18) nm; ^1^H NMR (500 MHz, CDCl_3_) and ^13^C NMR (125 MHz, CDCl_3_) shown in Table S1–S2; ESIMS *m/z* 417.2 [M + Na]^+^.

**TL-1-monoactate (10)**: yellow powder, [α]^25^_D_ −84.3 (*c* 0.3, CHCl_3_); ECD (*c* 0.76 mM, MeOH) λ_max_ (Δ*ε*) 238 (+0.38), 261 (−0.19), 311 (+2.96) and 392 (−1.36) nm; ^1^H NMR (500 MHz, CDCl_3_) and ^13^C NMR (125 MHz, CDCl_3_) shown in Table S1–S2; ESIMS *m/z* 415.2 [M + Na]^+^.

**Isochromophilone I (11)**: yellow powder, [α]^25^_D_ +89.7(*c* 0.6, EtOH); ECD (*c* 1.44 mM, MeOH) λ_max_ (Δ*ε*) 234 (+0.93), 264 (−0.46), 315 (+3.21) and 345 (+2.46) nm; ^1^H NMR (500 MHz, CDCl_3_) and ^13^C NMR (125 MHz, CDCl_3_) shown in Table S1 and S3; ESIMS *m/z* 417.2 [M + H]^+^.

**Ochrephilone(12)**: yellow powder, [α]^25^_D_ +89.4 (*c* 0.3, EtOH); ECD (*c* 0.78 mM, MeOH) λ_max_ (Δ*ε*) 238 (+3.02), 262 (−1.55), 338 (+15.33), 392 (−0.71) and 421 (+1.87) nm; ^1^H NMR (500 MHz, CDCl_3_) and ^13^C NMR (125 MHz, CDCl_3_) shown in Table S1 and S3; ESIMS *m/z* 383.2 [M + H]^+^.

**8-Acetyldechloroisochromophilone III (13)**: yellow powder, [α]^25^_D_ −105.8 (*c* 0.2, EtOH ); ECD (*c* 0.55 mM, MeOH) λ_max_ (Δ*ε*) 220 (+0.41), 244 (+2.59), 304 (−4.25) and 380 (+3.34) nm; ^1^H NMR (500 MHz, DMSO-*d*_6_,) and ^13^C NMR (125 MHz, DMSO-*d*_6_,) shown in Table S1 and S3; ESIMS *m/z* 361.3 [M + H]^+^.

**Scleratioramine (14)**: red powder, [α]^25^_D_ +253.4 (*c* 0.025, MeOH); ECD (*c* 1.28 mM, MeOH) λ_max_ (Δ*ε*) 214 (−3.87), 246 (+3.04) , 315 (−4.03) and 378 (+5.83) nm; IR (KBr) *ν*_max_ 3552, 2953, 2357, 1699, 1540, 1458, 1248 cm^-1^; ^1^H NMR (600 MHz, CDCl_3_) and ^13^C NMR (150 MHz, CDCl_3_) shown in Table S1 and S3; HRESIMS *m/z* 390.1472 [M + H]^+^ (calcd for C_21_H_25_O_4_NCl, 390.1467).

**Isochromophilone VI (15)**: red powder, [α]^25^_D_ +176.3 (*c* 0.025, MeOH); ECD (*c* 0.69 mM, MeOH) λ_max_ (Δ*ε*) 216 (−0.44), 244 (+6.96) , 304 (−10.78) and 381 (+8.85) nm; IR (KBr) *ν*_max_ 3370, 2961, 2357, 1699, 1591, 1500, 1230 cm^-1^; ^1^H NMR (600 MHz, CDCl_3_) and ^13^C NMR (150 MHz, CDCl_3_) shown in Table S1 and S3; HRESIMS *m/z* 434.1732 [M + H]^+^ (calcd for C_23_H_29_O_5_NCl, 434.1729).

**Isochromophilone IX (16)**: red powder, [α]^25^_D_ +194.9 (*c* 0.025, MeOH); ECD (*c* 0.84 mM, MeOH) λ_max_ (Δ*ε*) 216 (+0.22), 245 (+1.88) , 303 (−2.91) and 380 (+2.25) nm; IR (KBr) *ν*_max_ 2953, 2363, 1702, 1592, 1493, 1215, 1148, 759 cm^-1^; ^1^H NMR (600 MHz, CDCl_3_) and ^13^C NMR (150 MHz, CDCl_3_) shown in Table S1 and S3; HRESIMS *m/z* 476.1837 [M + H]^+^ (calcd for C_25_H_31_O_6_NCl, 476.1834).

**The Specific Rotation of Synthetic Compounds 4, 5 and 14–16**

**Synthetic 4**: red powder; [α]^25^_D_ +212.2 (*c* 0.025, MeOH);

**Synthetic 5**: red powder; [α]^25^_D_ +148.0 (*c* 0.025, MeOH);

**Synthetic 14**: red powder; [α]^25^_D_ +248.6 (*c* 0.025, MeOH);

**Synthetic 15**: red powder; [α]^25^_D_ +168.5 (*c* 0.025, MeOH);

**Synthetic 16**: red powder; [α]^25^_D_ +215.2 (*c* 0.025, MeOH).

**Figure S1**. The HRESIMS Spectrum of Compound **1**

^
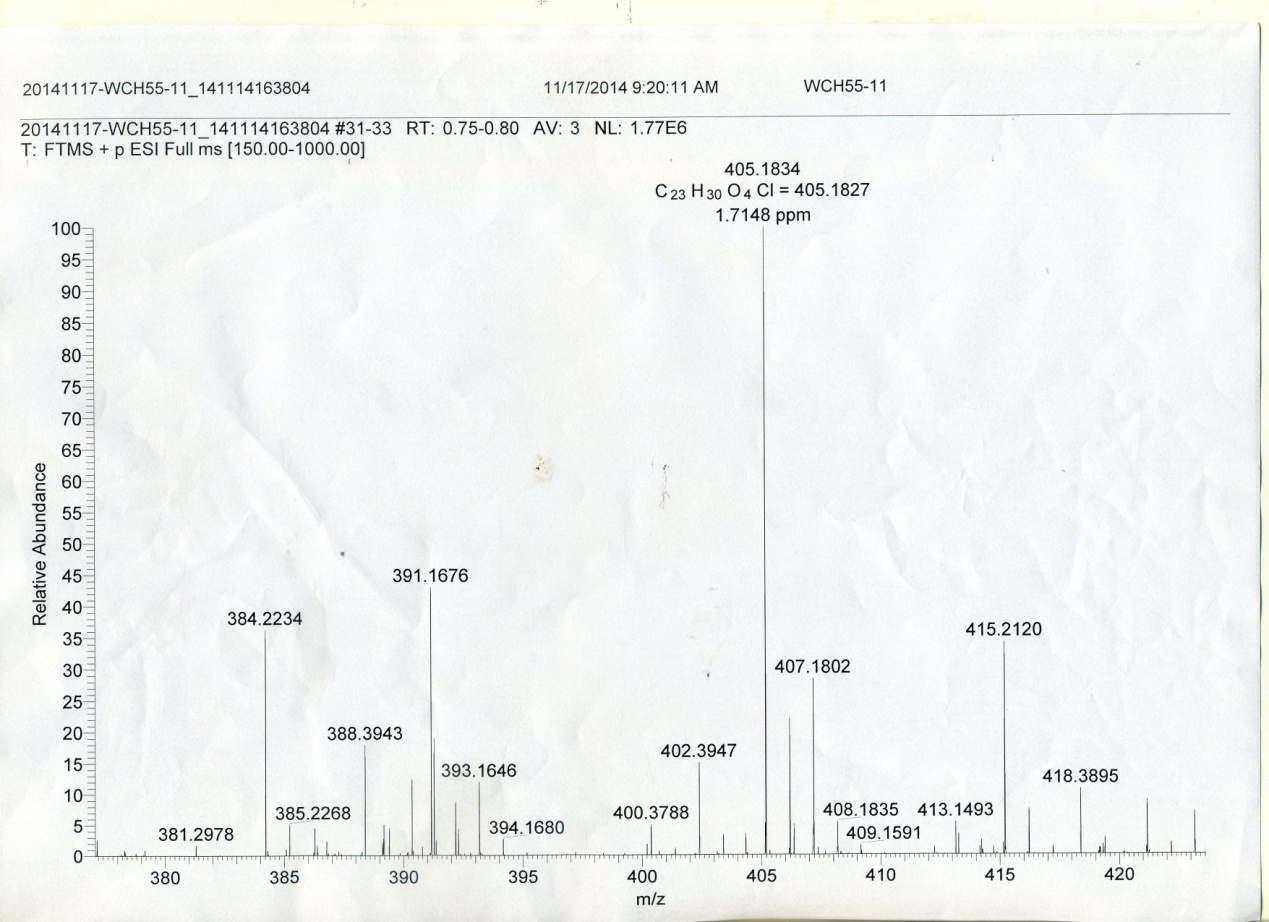
^

**Figure S2**. The ^1^H-NMR Spectrum of Compound **1** in DMSO-*d_6_*

**Figure S3**. The ^13^C-NMR Spectrum of Compound **1** in DMSO-*d_6_*

**Figure S4**. The DEPT Spectrum of Compound **1** in DMSO-*d_6_*

**Figure S5**. The HSQC NMR Spectrum of Compound **1** in DMSO-*d_6_*

**Figure S6**. The ^1^H-^1^H COSY Spectrum of Compound **1** in DMSO-*d_6_*

**Figure S7**. The HMBC Spectrum of Compound **1** in DMSO-*d_6_*

**Figure S8**. The NOESY Spectrum of Compound **1** in DMSO-*d_6_*

**Figure S9**. The NOE Difference Spectrum of Compound **1** in DMSO-*d_6_*

**Figure S10**. The HRESIMS Spectrum of Compound **2**

^
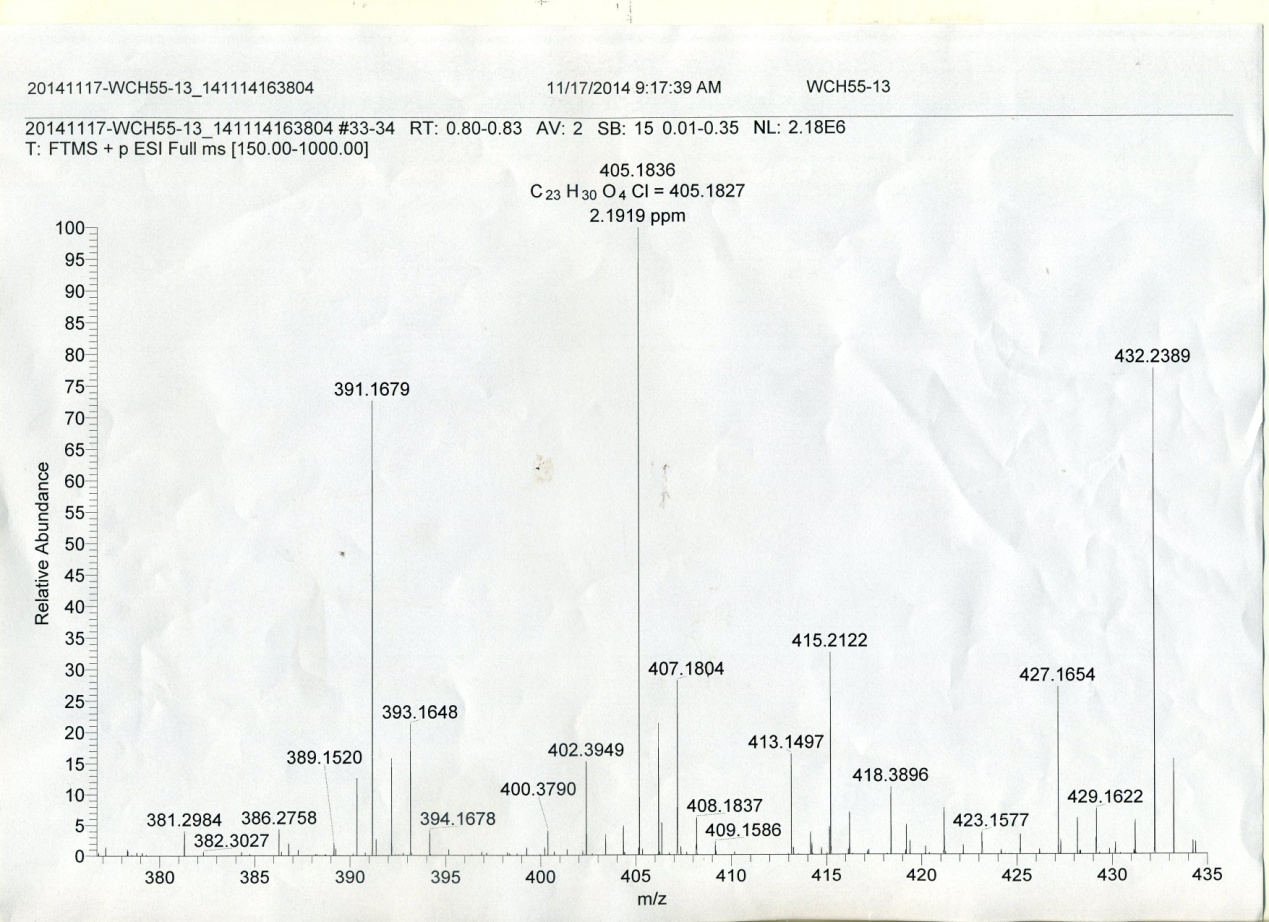
^

**Figure S11**. The ^1^H-NMR Spectrum of Compound **2** in DMSO-*d_6_*

**Figure S12**. The ^13^C-NMR Spectrum of Compound **2** in DMSO-*d_6_*

**Figure S13**. The DEPT Spectrum of Compound **2** in DMSO-*d_6_*

**Figure S14**. The HSQC Spectrum of Compound **2** in DMSO-*d_6_*

**Figure S15**. The ^1^H-^1^H COSY Spectrum of Compound **2** in DMSO-*d_6_*

**Figure S16**. The HMBC Spectrum of Compound **2** in DMSO-*d_6_*

**Figure S17**. The NOESY Spectrum of Compound **2** in DMSO-*d_6_*

**Figure S18**. The HRESIMS Spectrum of Compound **3**


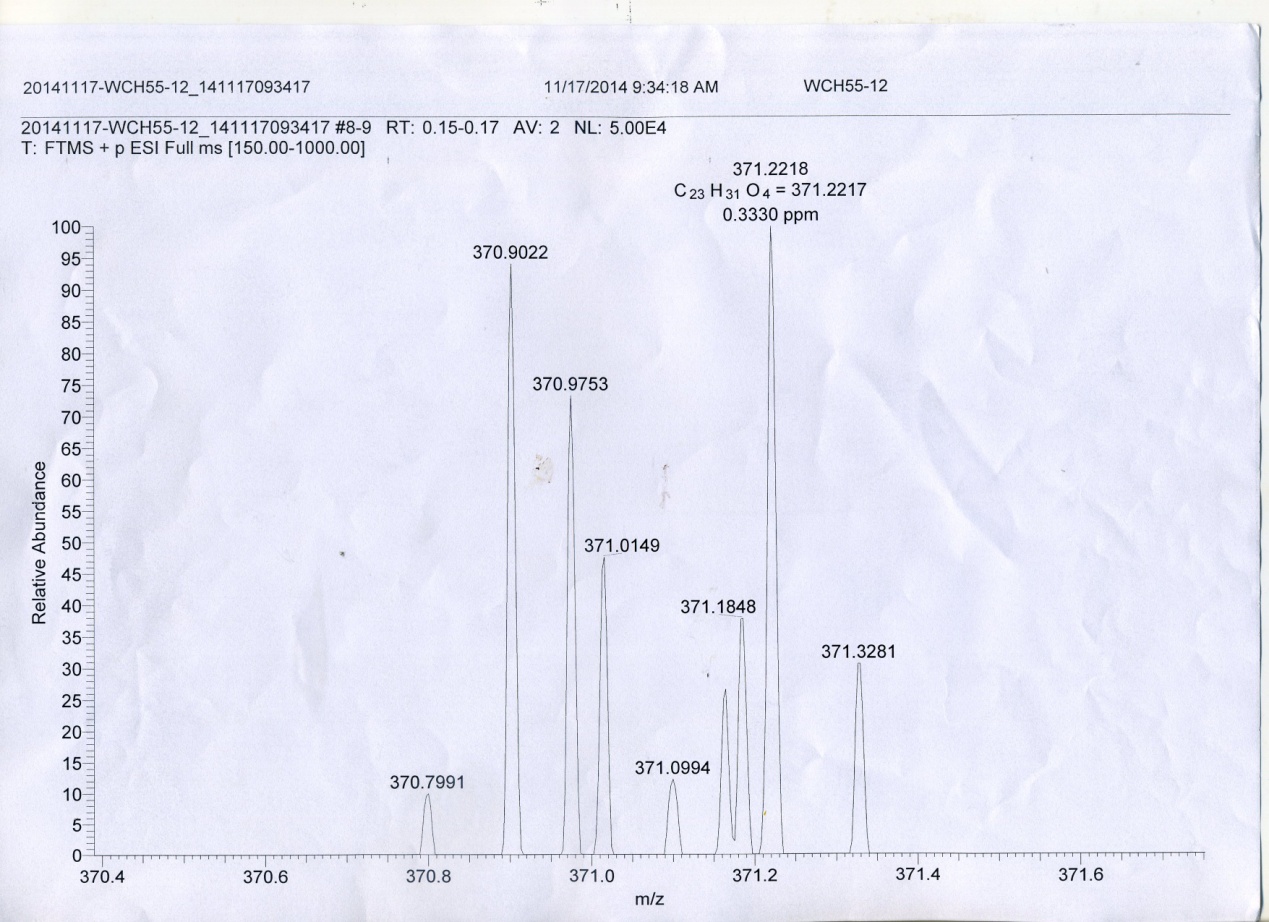


**Figure S19**. The ^1^H-NMR Spectrum of Compound **3** in DMSO-*d_6_*

**Figure S20**. The ^13^C-NMR Spectrum of Compound **3** in DMSO-*d_6_*

**Figure S21**. The DEPT Spectrum of Compound **3** in DMSO-*d_6_*

**Figure S22**. The HSQC Spectrum of Compound **3** in DMSO-*d_6_*

**Figure S23**. The ^1^H-^1^H COSY Spectrum of Compound **3** in DMSO-*d_6_*

**Figure S24**. The HMBC Spectrum of Compound **3** in DMSO-*d_6_*

**Figure S25**. The NOESY Spectrum of Compound **3** in DMSO-*d_6_*

**Figure S26**. The HRESIMS Spectrum of Compound **4**

**
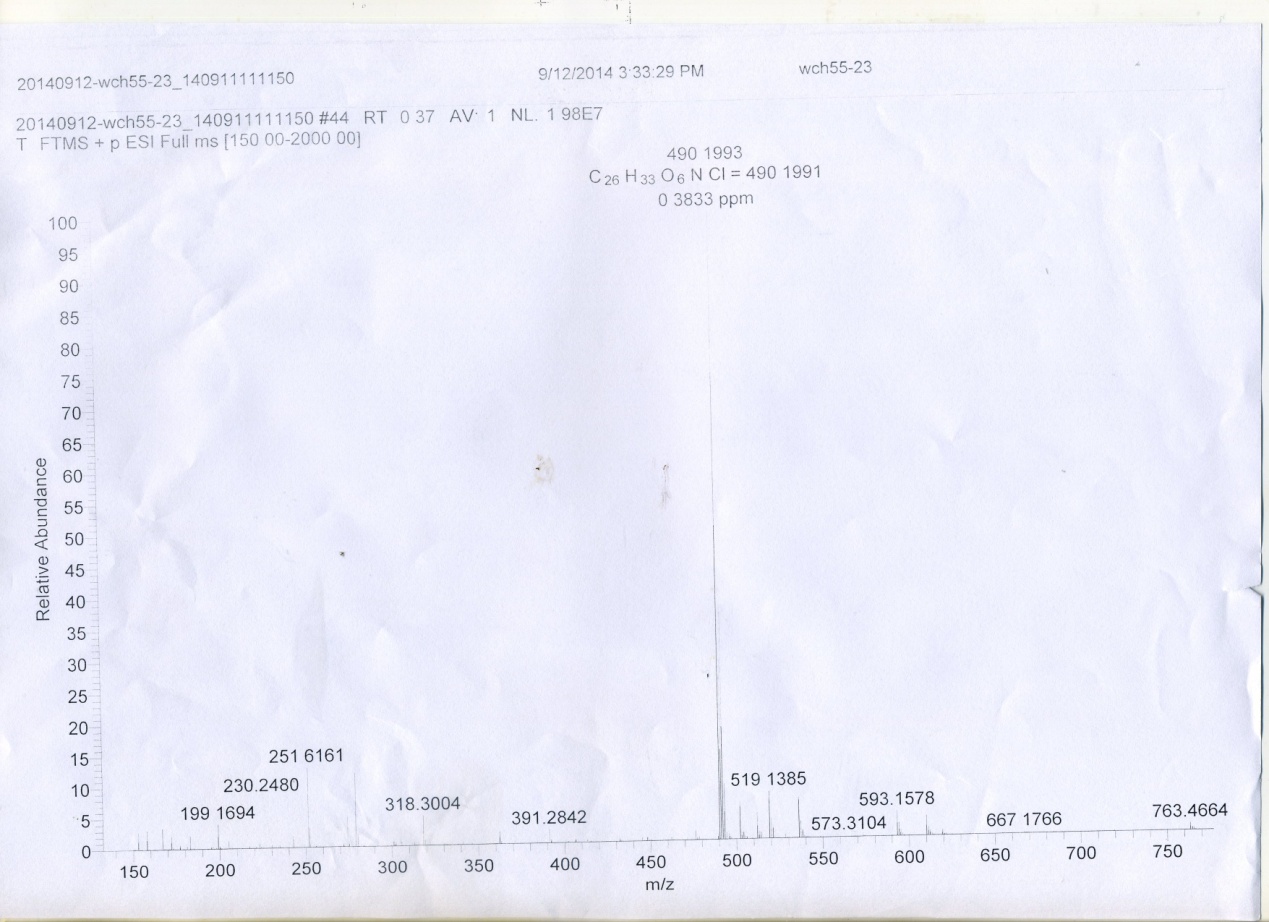
**

**Figure S27**. The ^1^H-NMR Spectrum of Compound **4** in CDCl_3_

**Figure S28**. The ^13^C-NMR Spectrum of Compound **4** in CDCl_3_

**Figure S29**. The DEPT Spectrum of Compound **4** in CDCl_3_

**Figure S30**. The HSQC Spectrum of Compound **4** in CDCl_3_

**Figure S31**. The ^1^H-^1^H COSY Spectrum of Compound **4** in CDCl_3_

**Figure S32**. The HMBC Spectrum of Compound **4** in CDCl_3_

**Figure S33**. The HRESIMS Spectrum of Compound **5**


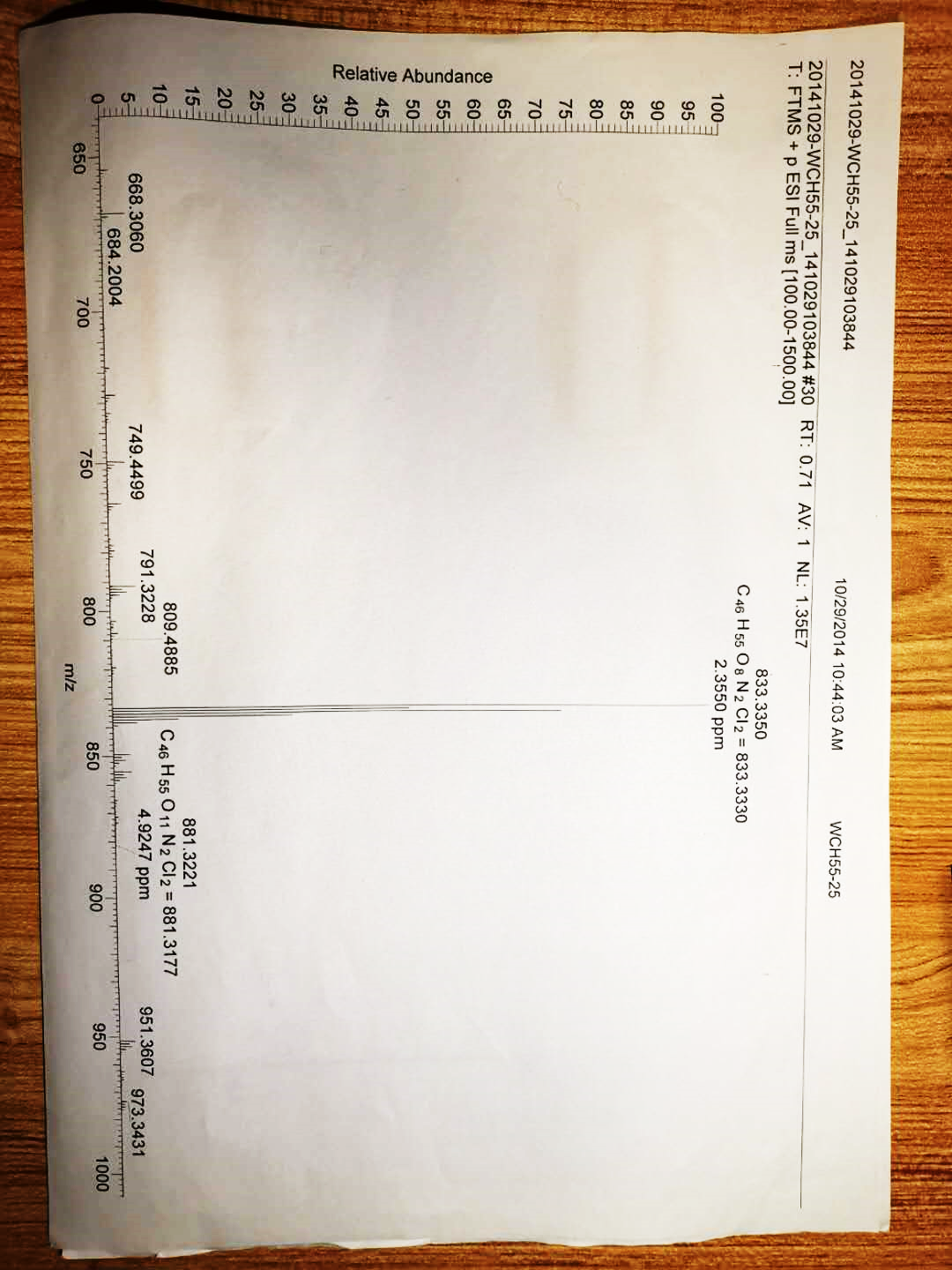


**Figure S34**. The ^1^H-NMR Spectrum of Compound **5** in CDCl_3_

**Figure S35**. The ^13^C-NMR Spectrum of Compound **5** in CDCl_3_

**Figure S36**. The DEPT Spectrum of Compound **5** in CDCl_3_

**Figure S37**. Co-HPLC profiles of the synthetic and the natural **4**, **5** and **14**−**16**


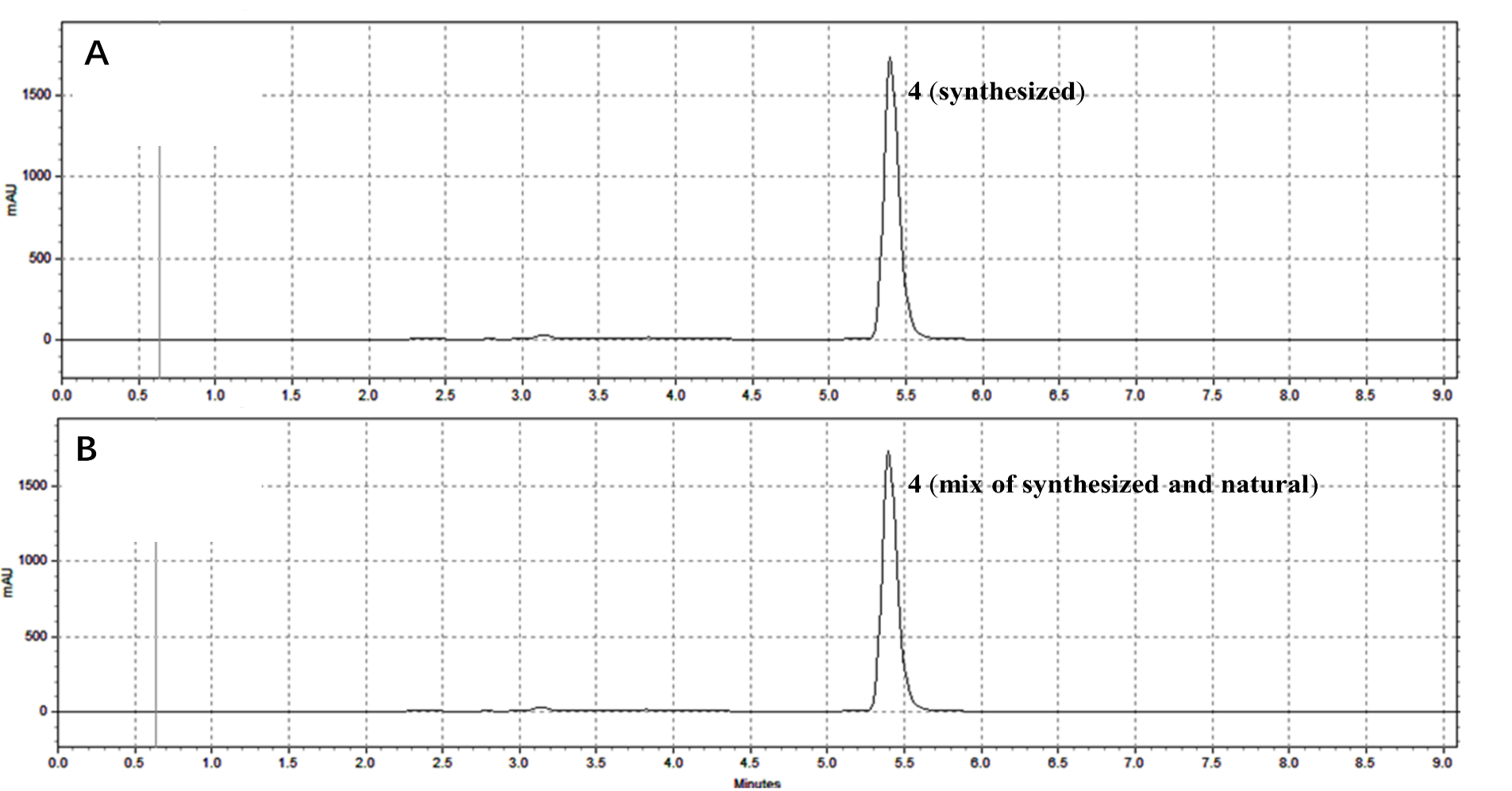


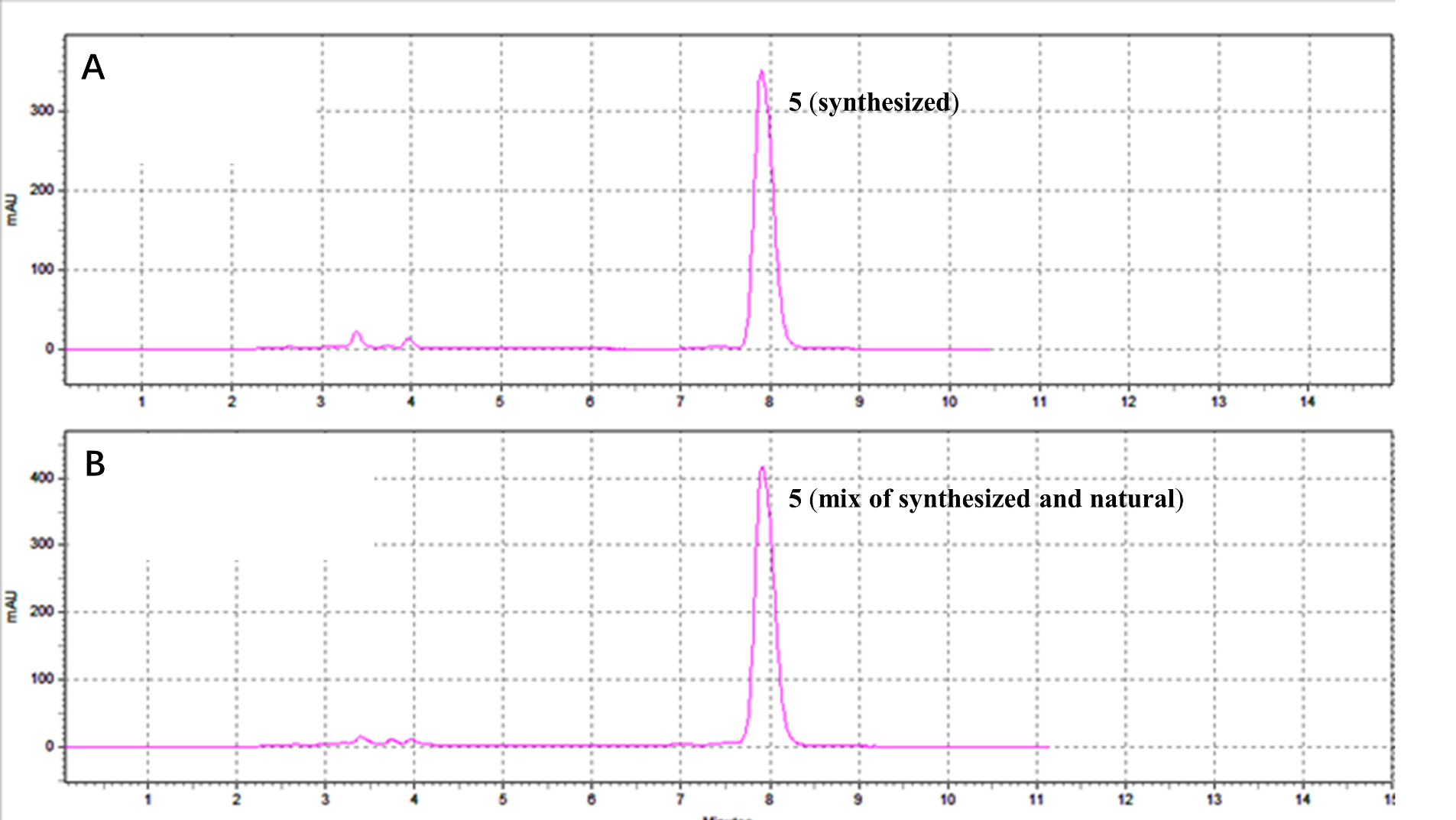

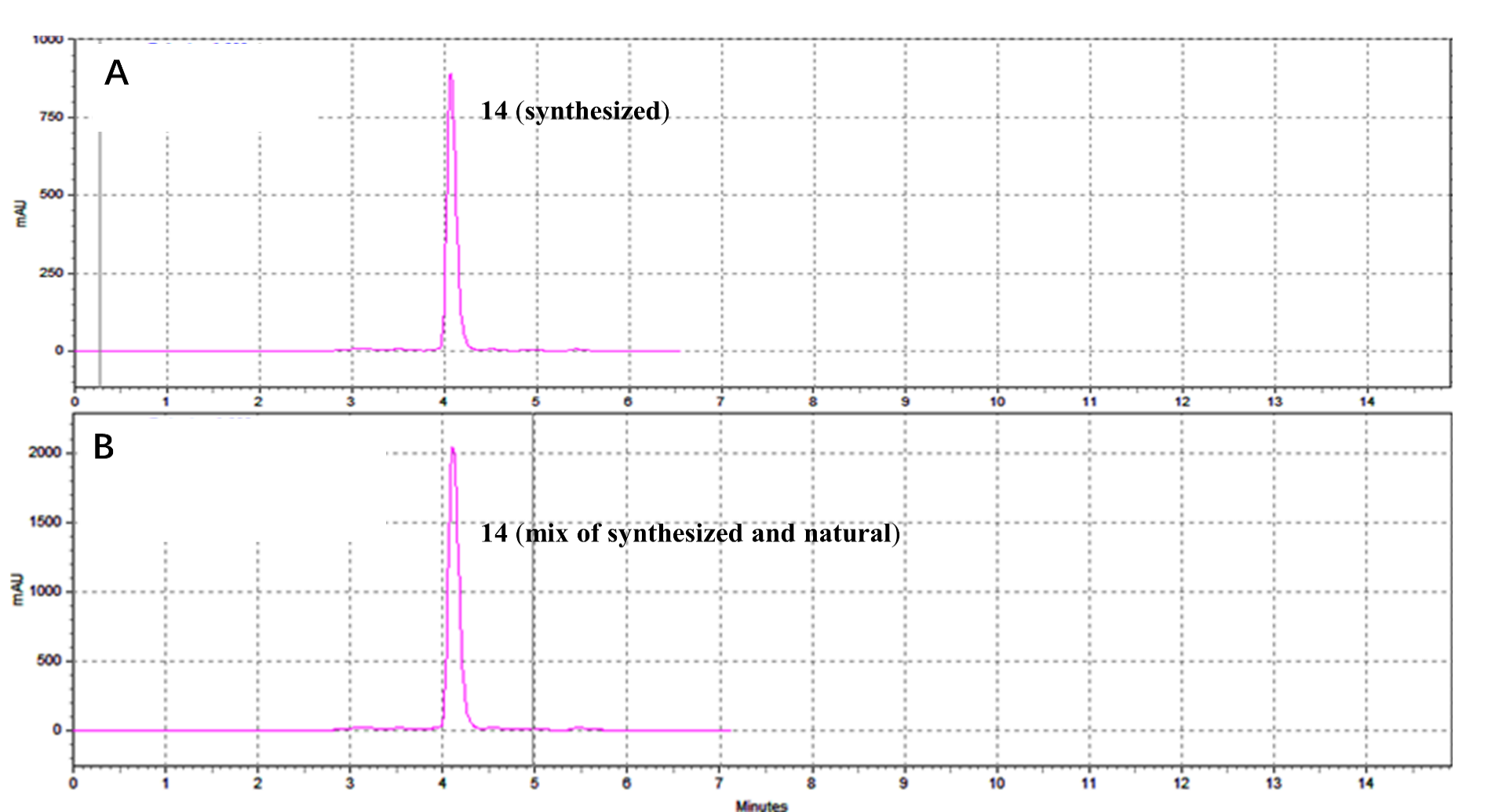


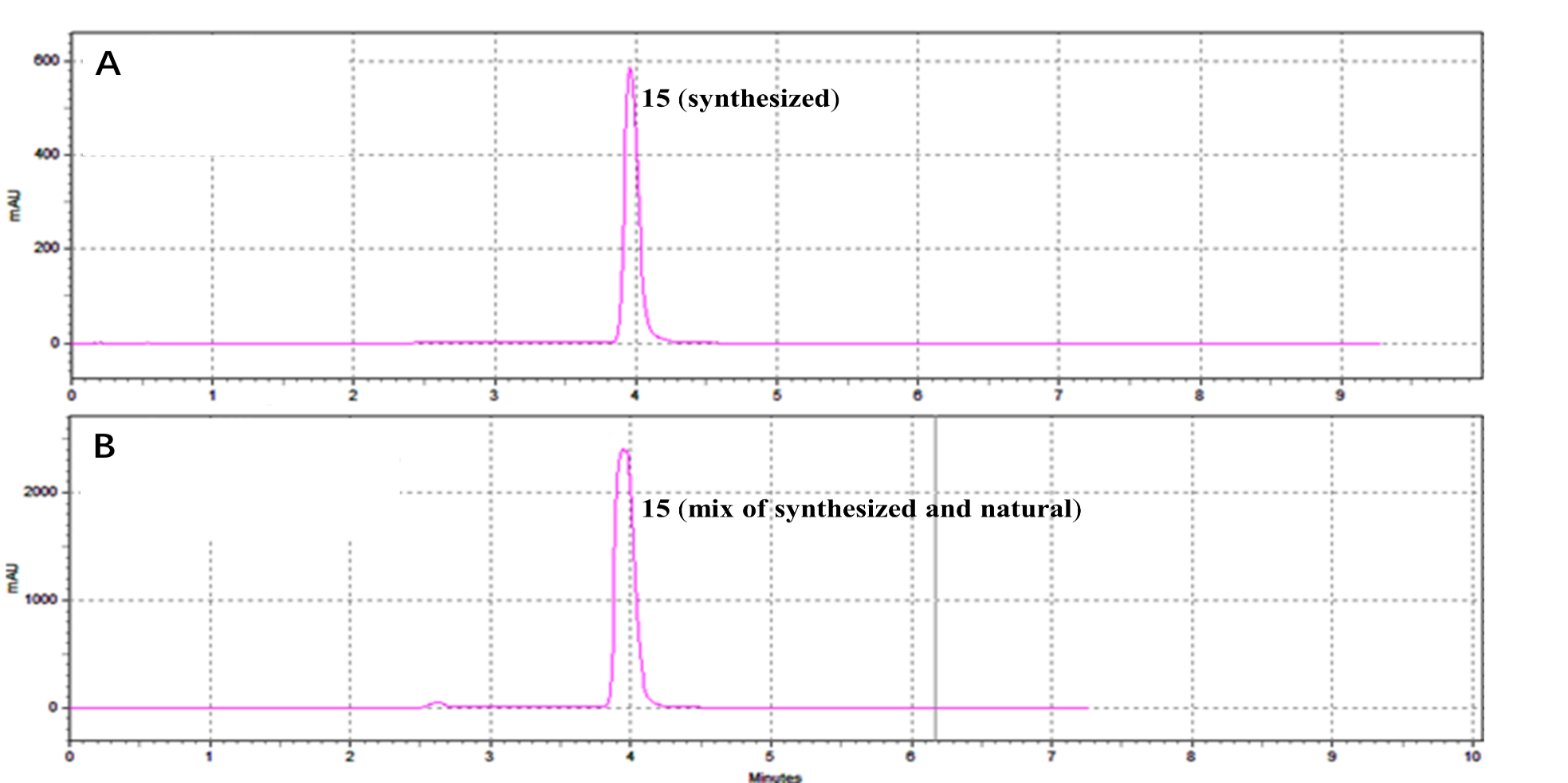


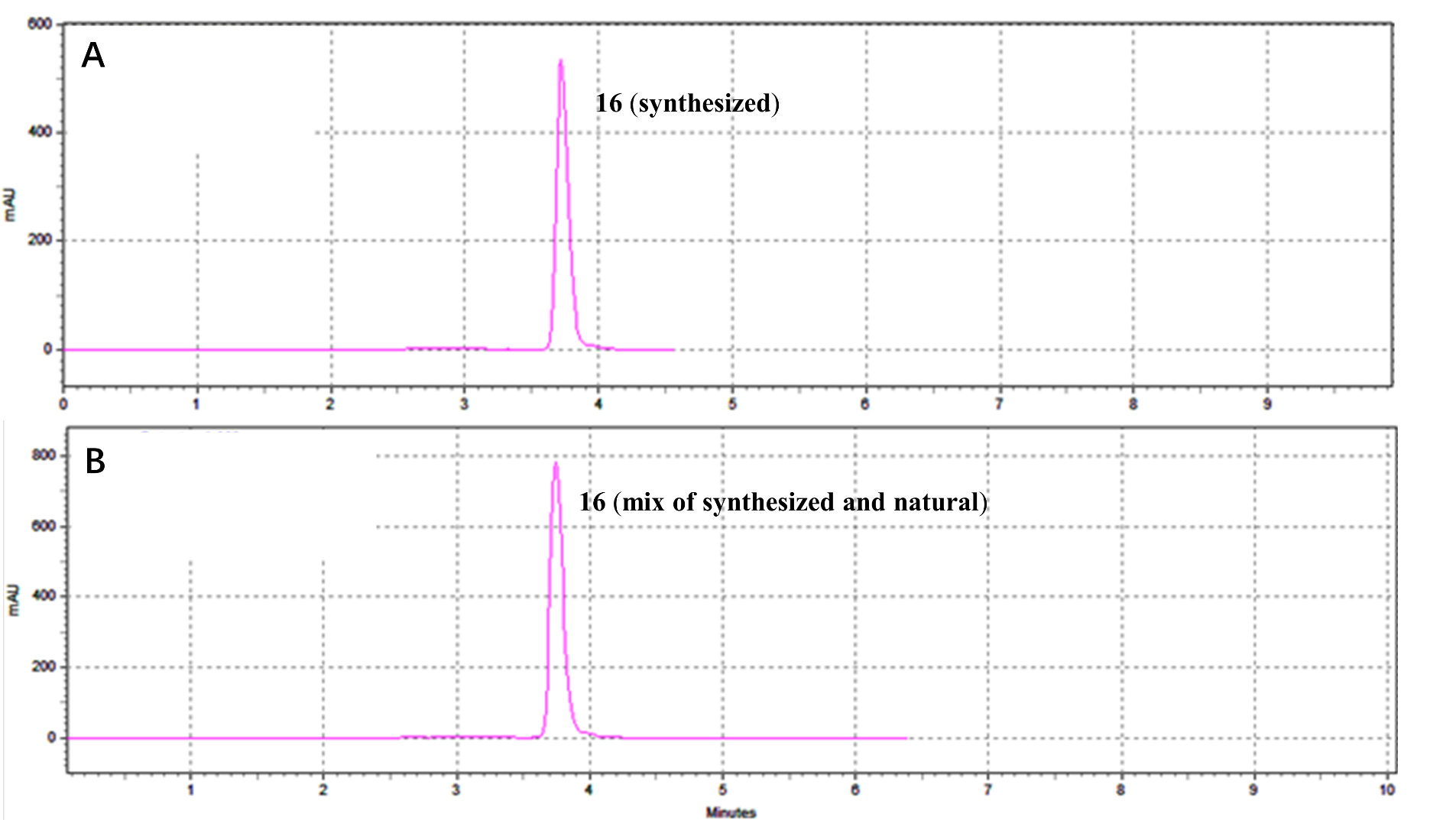


HPLC profiles of the synthetic **4**, **5** and **14**–**16** co-eluted with the corresponding natural ones on an ODS column (flow rate: 1 mL/min; solvent: 85% MeCN/H_2_O; detection: 339 nm; temperature: 30 °C). (**A**) HPLC profile of the synthetic **4**, **5** and **14**–**16**; (**B**) mixed HPLC profile of the synthetic **4**, **5** and **14**–**16** with the corresponding natural **4**, **5** and **14**–**16**.

**Figure S38**. Measured ECD curves of compounds **4**, **5**, **7** and **14**−**16**.





**Figure S39**. HPLC of the reaction products from **14** to **5**


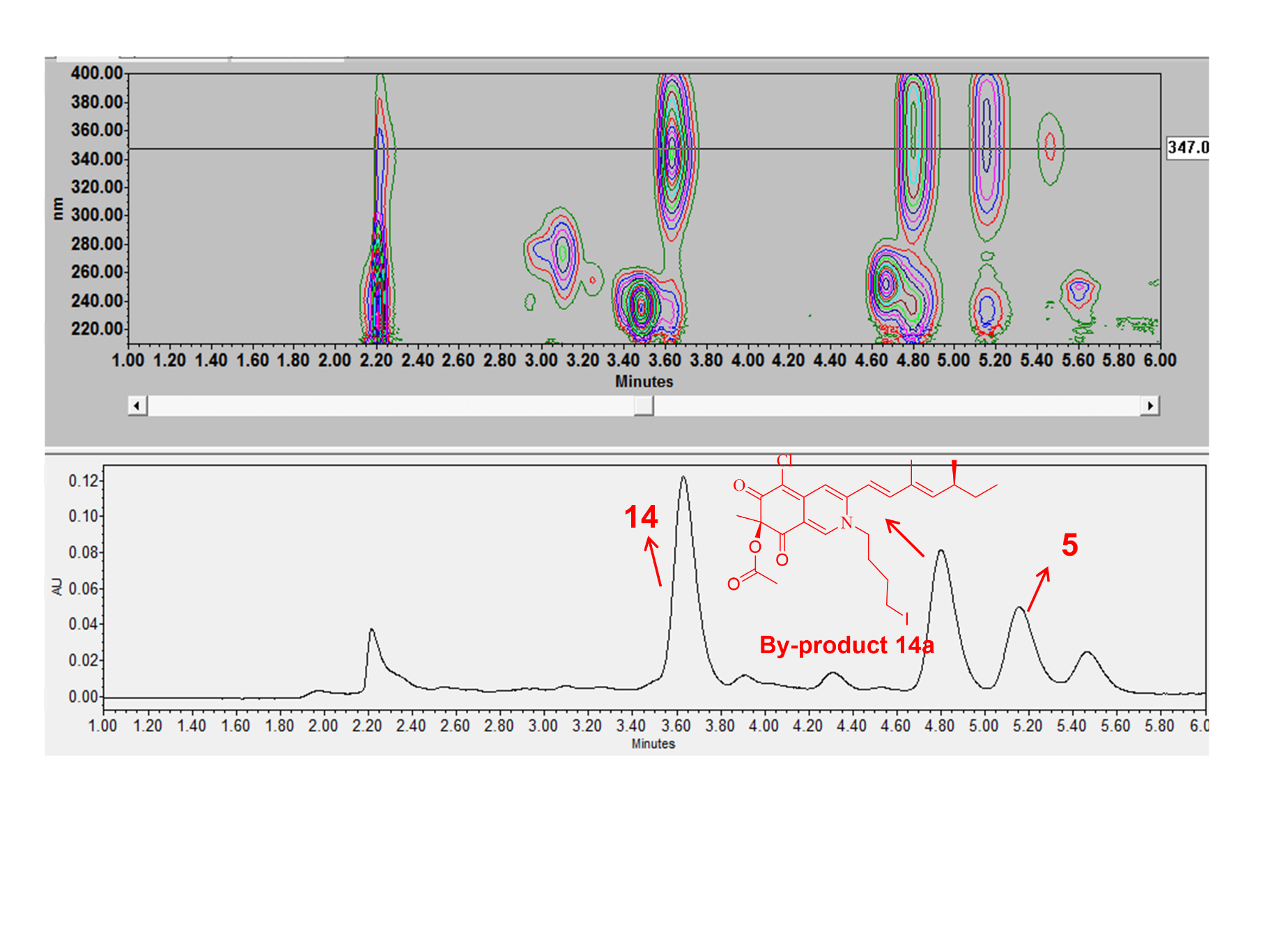


HPLC profiles of the reaction products from **14** to **5** on Cosmosil packed column (flow rate: 1 mL/min; solvent: 90% Acetonitrile-H_2_O/0.5‰ CF_3_COOH; detection: 347 nm; temperature: 30 °C).
